# Angiotensin-(1-7)/MasR axis promotes migration of monocytes/macrophages with a regulatory phenotype to perform phagocytosis and efferocytosis

**DOI:** 10.1172/jci.insight.147819

**Published:** 2022-01-11

**Authors:** Isabella Zaidan, Luciana P. Tavares, Michelle A. Sugimoto, Kátia M. Lima, Graziele L. Negreiros-Lima, Lívia C.R. Teixeira, Thais C. Miranda, Bruno V.S. Valiate, Allysson Cramer, Juliana Priscila Vago, Gabriel H. Campolina-Silva, Jéssica A.M. Souza, Laís C. Grossi, Vanessa Pinho, Maria Jose Campagnole-Santos, Robson A.S. Santos, Mauro M. Teixeira, Izabela Galvão, Lirlândia P. Sousa

**Affiliations:** 1 Signaling in Inflammation Laboratory, Department of Clinical and Toxicological Analysis, Faculty of Pharmacy, Federal University of Minas Gerais, Belo Horizonte, Brazil.; 2 Immunopharmacology Laboratory, Department of Biochemistry and Immunology, Institute of Biological Sciences, Federal University of Minas Gerais, Belo Horizonte, Brazil.; 3 Division of Pulmonary and Critical Care Medicine, Department of Medicine, Brigham and Women’s Hospital, Boston, Massachusetts, USA.; 4 Department of Morphology and; 5 Department of Physiology and Pharmacology, Institute of Biological Sciences, Federal University of Minas Gerais, Belo Horizonte, Brazil.

**Keywords:** Infectious disease, Inflammation, Bacterial infections, Cell migration/adhesion, Macrophages

## Abstract

Nonphlogistic migration of macrophages contributes to the clearance of pathogens and apoptotic cells, a critical step for the resolution of inflammation and return to homeostasis. Angiotensin-(1-7) [Ang-(1-7)] is a heptapeptide of the renin-angiotensin system that acts through Mas receptor (MasR). Ang-(1-7) has recently emerged as a novel proresolving mediator, yet Ang-(1-7) resolution mechanisms are not fully determined. Herein, Ang-(1-7) stimulated migration of human and murine monocytes/macrophages in a MasR-, CCR2-, and MEK/ERK1/2–dependent manner. Pleural injection of Ang-(1-7) promoted nonphlogistic mononuclear cell influx alongside increased levels of CCL2, IL-10, and macrophage polarization toward a regulatory phenotype. Ang-(1-7) induction of CCL2 and mononuclear cell migration was also dependent on MasR and MEK/ERK. Of note, MasR was upregulated during the resolution phase of inflammation, and its pharmacological inhibition or genetic deficiency impaired mononuclear cell recruitment during self-resolving models of LPS pleurisy and *E*. *coli* peritonitis. Inhibition/absence of MasR was associated with reduced CCL2 levels, impaired phagocytosis of bacteria, efferocytosis, and delayed resolution of inflammation. In summary, we have uncovered a potentially novel proresolving feature of Ang-(1-7), namely the recruitment of mononuclear cells favoring efferocytosis, phagocytosis, and resolution of inflammation. Mechanistically, cell migration was dependent on MasR, CCR2, and the MEK/ERK pathway.

## Graphical abstract

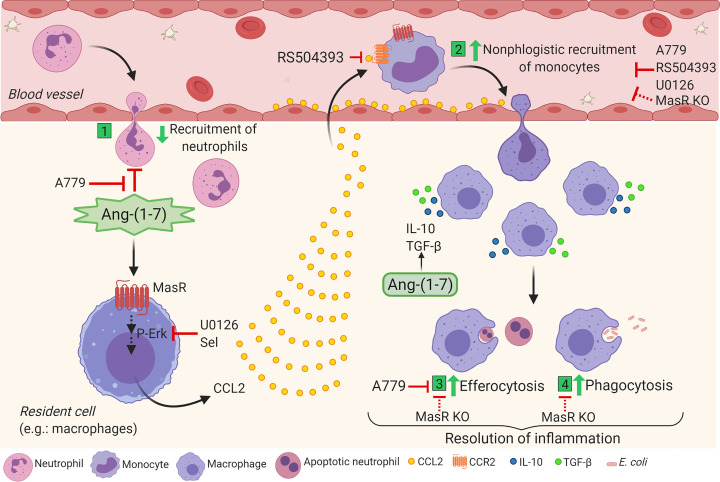

## Introduction

The renin-angiotensin system (RAS) is traditionally viewed as an enzymatic cascade of reactions that culminate with the production of angiotensin II (Ang II), a hormone peptide that causes vasoconstriction and is endowed with potent proinflammatory actions (1). RAS activation is often associated with the pathological induction of inflammatory and profibrotic responses in different diseases (2, 3). Conversely, an alternative counterregulatory branch of the RAS was later described: the angiotensin-converting enzyme 2/angiotensin-(1-7)/Mas receptor axis. Angiotensin-(1-7) [Ang-(1-7)] is a bioactive heptapeptide produced through the enzymatic inactivation of Ang II by the angiotensin-converting enzyme 2 (ACE-2) (4, 5). In addition to directly inhibiting Ang II, the production and bioactions of Ang-(1-7) include potent receptor-mediated antiinflammatory responses triggered by the activation of the G protein–coupled receptor Mas (MasR) (6). Accordingly, MasR absence is associated with the aggravation of cardiovascular and inflammatory diseases (7–10).

The Ang-(1-7)/MasR axis was recently shown to mediate important features of resolution of inflammation, including apoptosis of neutrophils with subsequent efferocytosis, and decrease the secretion of proinflammatory cytokine and granulocyte recruitment (11–13). Nevertheless, the compelling tissue-protective and regulatory actions of Ang-(1-7) remain poorly explored. Understanding the specific proresolving mechanisms of Ang-(1-7) in leukocyte responses will shed light on critical therapeutic targets for inflammatory diseases.

Macrophages are key leukocytes promoting both inflammation onset and resolution (14, 15). The plasticity of macrophage response is evidenced by the distinct functional cell phenotypes determined by different tissue milieus (15, 16). Upon injury, activated macrophages rapidly produce significant amounts of cytokines, chemokines, and other mediators that promote inflammation. Once the inflammatory trigger is eliminated, macrophage phenotype is switched toward regulatory profiles and is more prone to perform efferocytosis of apoptotic cells and secrete antiinflammatory and proresolving mediators. Accumulating evidence indicates that the Ang-(1-7)/MasR axis deactivates proinflammatory macrophages (9, 12, 17, 18). For instance, MasR is expressed in different macrophage subtypes and negatively regulates the production of phlogistic mediators (8, 9). Of note, the MasR ligand Ang-(1-7) significantly counterregulates LPS-induced proinflammatory macrophage responses (12, 17, 18). Although the role of Ang-(1-7)/MasR in the deactivation of proinflammatory macrophages has already been shown, the mechanisms by which Ang-(1-7) promotes nonphlogistic monocyte/macrophage migration, laying the foundation for inflammation resolution, remain unknown.

Here, we identified a potentially novel proresolving feature of Ang-(1-7), namely the nonphlogistic recruitment of macrophages. Ang-(1-7) signaling promoted macrophage migration, polarization to regulatory phenotypes, efferocytosis, and phagocytosis and induced resolution of inflammation through its cognate receptor, Mas, in models of sterile inflammation and infection. Mechanistically, Ang-(1-7)–induced cell migration alongside IL-10 and TGF-β release was dependent on the CCL2/CCR2 axis and activation of the MEK/ERK1/2 pathway.

## Results

### Ang-(1-7) promotes migration of macrophages and monocytes but not neutrophils.

**Figure 1:**
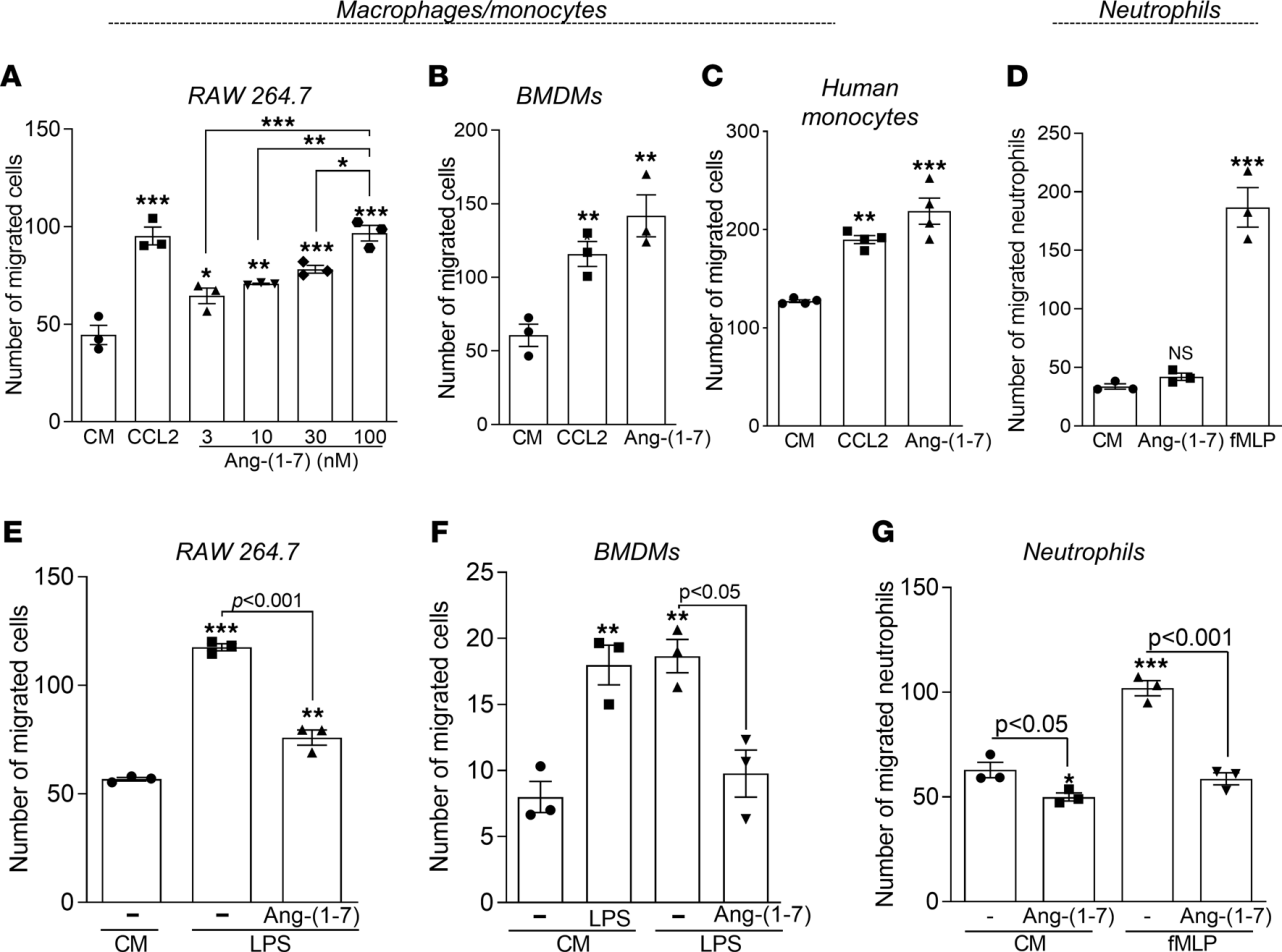
Macrophage and neutrophil chemotaxis after Ang-(1-7) treatment. RAW 264.7 cells (**A**), BMDMs (**B**), and human monocytes (**C**) were seeded in 24-transwell plates and allowed to migrate across a polycarbonate membrane using increasing concentrations of Ang-(1-7) as a chemoattractant (3-100 nM). CCL2 (100 ng) was used as a positive control. Chemotaxis assays using isolated human neutrophils were also performed using Ang-(1-7) (100 nM) or fMLP (10^–9^ M) as chemoattractants (**D**). Pretreatment of BMDMs (**E**), RAW 264.7 cells (**F**), and human neutrophils (**G**) for 1 hour with Ang-(1-7) 100 mM or media (RPMI) was performed to assess cell chemotaxis toward LPS (100 ng/mL) or fMLP (10^–9^ M/well) or control medium (CM) as specified in the graphs. Results are expressed as the number of migrated cells and are presented as mean ± SEM; * for *P* < 0.05, ** for *P* < 0.01, and *** for *P* < 0.001 when compared with the control group (CM) by 1-way ANOVA. Graphs **A**, **B**, **E**, and **F** are representative results of 3 independent experiments performed in biological triplicates (*n* = 3). Graphs **C**, **D**, and **G** represent data collected from different healthy volunteers (*n* = 4 for **C** and *n* = 3 for **D** and **G**).

To explore the role of Ang-(1-7) on macrophage migration, we initially performed in vitro chemotaxis assays using RAW 246.7 macrophages. Ang-(1-7) induced a concentration-dependent macrophage migration when compared with untreated cells (Figure 1A). A concentration as low as 3 nM of Ang-(1-7) promoted significant macrophage chemotaxis, which was further raised by increasing concentrations of the peptide (Figure 1A). In addition, Ang-(1-7) induced in vitro migration of BM-derived macrophages (BMDMs) and human monocytes (Figure 1, B and C) to the same extent as the classical monocyte chemoattractant, CCL2. In contrast to its action on macrophages, Ang-(1-7) did not induce chemotaxis of human neutrophils, as observed for the positive control (*N*-formylmethionyl-leucyl-phenylalanine — fMLP) (Figure 1D).

Next, we questioned whether pretreatment of macrophages and neutrophils with Ang-(1-7) would affect the in vitro migration toward phlogistic chemoattractants, mimicking cell recruitment in an inflammatory milieu. As anticipated, pre-exposure to Ang-(1-7) decreased the migration of macrophages (RAW 264.7 and BMDMs) and neutrophils toward the phlogistic stimuli LPS and fMLP, respectively (Figure 1, E–G). Notably, pretreatment of neutrophils with Ang-(1-7) decreased spontaneous migration of human neutrophils (toward control medium) (Figure 1G). Collectively, we have observed that monocyte/macrophages, but not neutrophils, migrated toward Ang-(1-7) in nonphlogistic conditions. In contrast, migration of neutrophils or macrophages toward inflammatory stimuli was prevented by pre-exposure to Ang-(1-7).

### Ang-(1-7)–induced monocyte/macrophage migration is dependent on MasR, CCR2, and MEK/ERK1/2 pathway.

**Figure 2:**
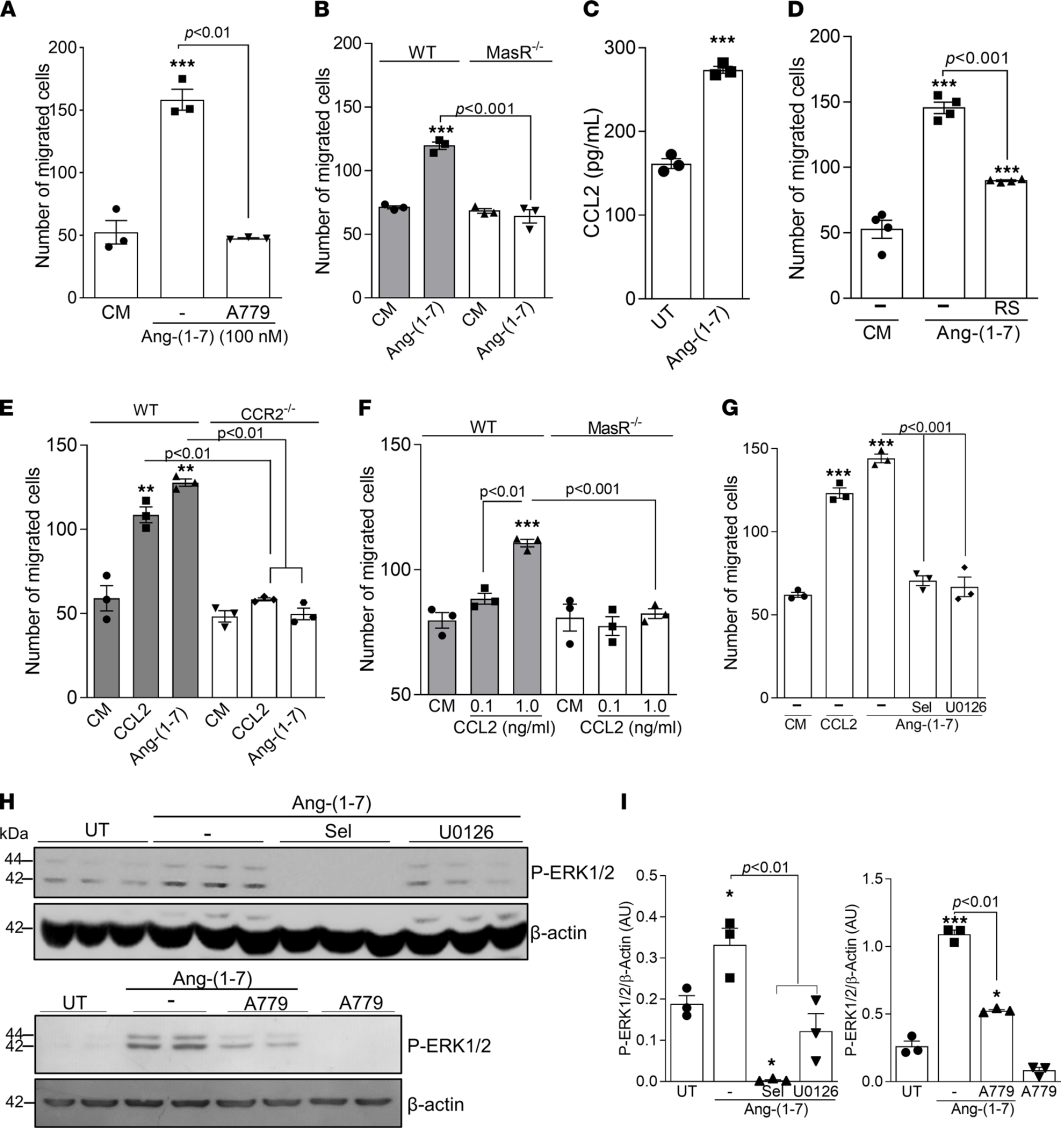
Ang-(1-7)–induced macrophage migration is dependent on MasR and associated with CCR2 and the MEK/ERK1/2 pathway. (**A**) RAW 264.7 cells were pretreated with the antagonist of MasR (A779, 1 μM) 1 hour before performing chemotaxis assays using Ang-(1-7) (100 nM) as the chemoattractant. (**B**) BMDMs from WT and MasR^–/–^ mice were next used to assess cell chemotaxis toward Ang-(1-7). (**C**) Graph shows the production of CCL2 by WT BMDMs 4 hours post–Ang-(1-7) exposure. (**D**) RAW 264.7 cells were pretreated with CCR2 antagonist (RS504393 10 μM) 1 hour before performing chemotaxis assays using Ang-(1-7) (100 nM) as the chemoattractant. (**E**) BMDMs from WT and CCR2^–/–^ mice were used to assess cell chemotaxis toward Ang-(1-7), and (**F**) a chemotaxis assay toward CCL2 (0.1 and 1 ng/mL) was performed using BMDMs from WT and MasR^–/–^ mice. (**G**) RAW 264.7 cells were pretreated for 1 hour with inhibitors of the MEK/ERK pathway (selumetinib and U0126, 10 and 15 μM, respectively), and chemotaxis assays using Ang-(1-7) (100 nM) as the chemoattractant were performed. (**H**) Western blot analyses for p-ERK1/2 were performed after incubation of RAW 264.7 cells with the abovementioned inhibitors followed by Ang-(1-7) treatment. β-Actin was used as a protein load control. (**I**) Quantification of blot bands was performed using ImageJ (NIH). Data are presented as mean ± SEM, * for *P* < 0.05, ** for *P* < 0.01, and *** for *P* < 0.001 when compared with the control group by 1-way ANOVA (**A**, **B**, and **D**–**I**) or 2-tailed *t* test (**C**). Data are representative of 3 independent experiments performed in biological triplicates or quadruplicates (*n* = 3–4). CM, control medium; UT, untreated cells.

Since Ang-(1-7) bioactions are mainly translated by MasR binding (6), we next evaluated the involvement of MasR in the Ang-(1-7)–induced macrophage migration. Pretreatment of RAW 264.7 macrophages with the antagonist of MasR, A779, abolished Ang-(1-7)–induced migration (Figure 2A). To validate our findings, we performed the migration assay using BMDMs from wild-type (WT) and MasR-knockout (MasR^–/–^) mice. Likewise, MasR deficiency abrogated Ang-(1-7)–induced migration of murine macrophages (Figure 2B).

Monocyte/macrophage migration largely relies on the CCL2/CCR2 pathway (19). Importantly, proresolving molecules, such as plasmin and db-cAMP, significantly stimulate CCR2-dependent macrophage migration (16, 20). Here, Ang-(1-7) induced a significant production of CCL2 by macrophages (Figure 2C), and pretreatment with RS504393 (CCR2 antagonist) significantly reduced macrophage chemotaxis toward Ang-(1-7) (Figure 2D). In agreement with that, BMDMs from CCR2^–/–^ mice presented no migration toward either CCL2 or Ang-(1-7) (Figure 2E). Of note, whereas BMDMs from WT mice efficiently migrated toward low concentrations of CCL2 (1 ng/mL), a complete absence of migration toward a CCL2 gradient was observed for MasR^–/–^ BMDMs (Figure 2F).

Next, we assessed the involvement of the MEK/ERK pathway in Ang-(1-7)–induced macrophage migration. MEK/ERK signaling is part of the cascade for cell migration in different contexts, including during resolution of inflammation (20, 21). Initial experiments revealed that Ang-(1-7) induced ERK1/2 phosphorylation — the kinase downstream to MEK (Supplemental Figure 1, A and B; supplemental material available online with this article; https://doi.org/10.1172/jci.insight.147819DS1) — and pretreatment of macrophages with 2 MEK/ERK inhibitors, selumetinib and U0126, decreased Ang-(1-7)–induced phosphorylated (p-) ERK1/2 and macrophage migration (Figure 2, G–I). Activation of ERK1/2 by Ang-(1-7) was partially dependent on MasR, as A779 significantly decreased P-ERK1/2 levels in Ang-(1-7)–treated macrophages (Figure 2, H and I).

### Ang-(1-7)/MasR axis promotes monocyte/macrophage migration in vivo.

**Figure 3:**
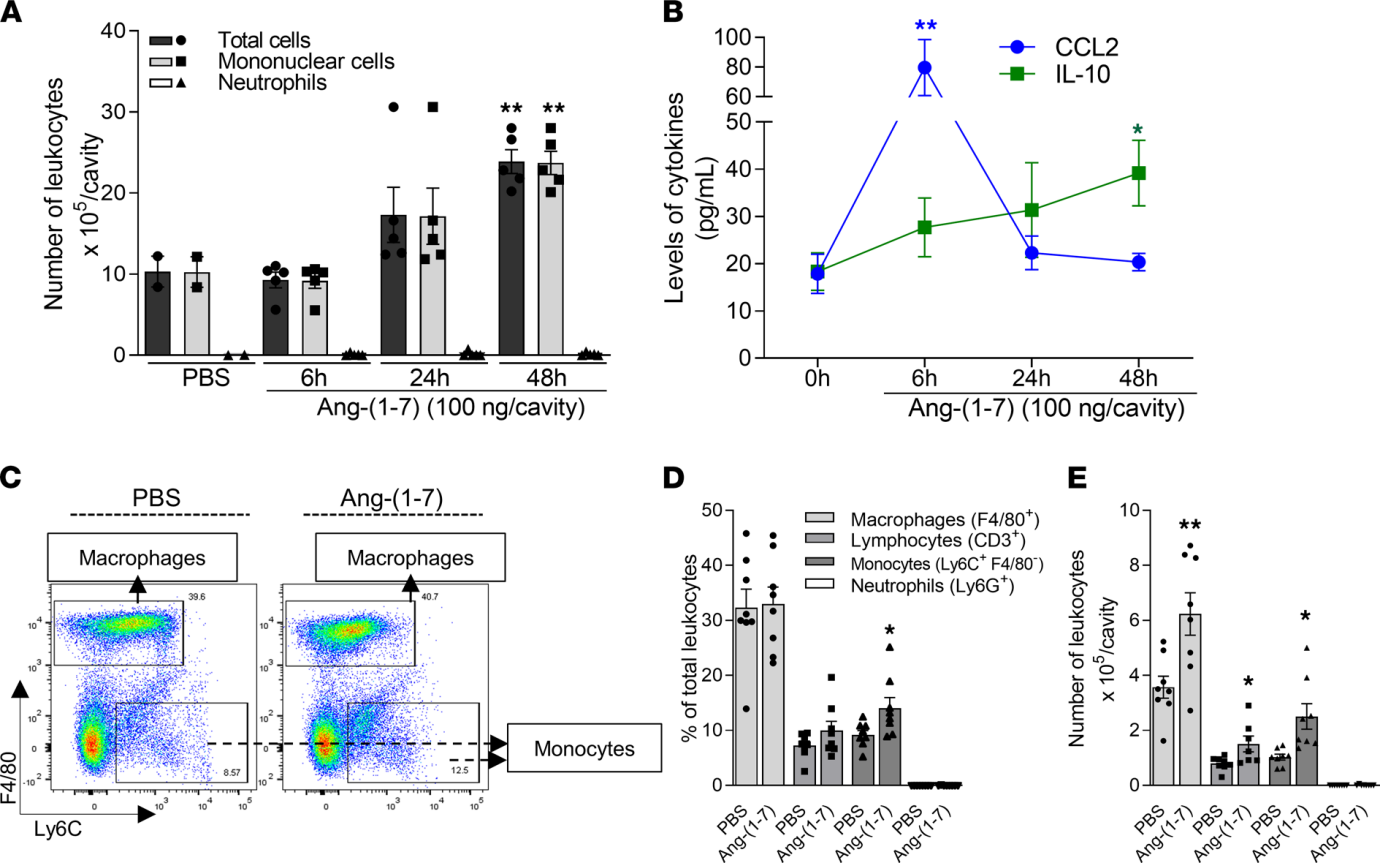
Ang-(1-7) induces time-dependent recruitment of monocytes/macrophages to the pleura of mice and increases IL-10 production. BALB/c mice received an intrapleural (i.pl.) injection of Ang-(1-7) (100 ng/cavity) or PBS (controls), and the cells recruited to the cavity were harvested at 6, 24, and 48 hours for total and differential leukocyte counts by light microscopy (**A**). Levels of CCL2 and IL-10 were measured by ELISA in the pleural lavage supernatants after PBS or Ang-(1-7) injections (**B**). Flow cytometry analysis of recruited leukocytes harvested 48 hours after Ang-(1-7) or PBS injection was also performed. Representative dot plots (**C**), leukocyte frequencies (expressed as the percentage of single cells), and leukocyte numbers are presented (**D** and **E**). Results are presented as mean ± SEM (graphs **A** *n* = 5, **B** *n* = 6, **D** and **E** *n* = 8), * for *P* < 0.05 and ** for *P* < 0.01 when compared with control (PBS), by 1-way ANOVA.

Having established the role of Ang-(1-7) in macrophage migration in vitro, we next examined Ang-(1-7) actions in vivo. Ang-(1-7) induced a time-dependent influx of leukocytes into the pleural cavity of mice (Figure 3A). Leukocyte infiltration was almost entirely composed of mononuclear cells without any significant recruitment of neutrophils. Likewise, Ang-(1-7) injected in the intra-articular cavity of mice induced a similar profile of leukocyte influx into the knee joint of mice (peak at 24 hours) (Supplemental Figure 2A).

Ang-(1-7)–induced cell recruitment into the pleural cavity was associated with increased production of IL-10 and CCL2 (Figure 3B) without modifying the levels of CXCL1 and proinflammatory cytokines, such as TNF-α and IL-6 (Supplemental Figure 2C). Similarly, increased CCL2 levels were associated with the recruitment of cells into the intra-articular cavity of mice post–Ang-(1-7) injection (Supplemental Figure 2B).

Additional immunophenotyping of cells recruited into the pleura showed enrichment in the frequency of monocytes 48 hours post–Ang-(1-7) (Figure 3, C and D) along with increased absolute numbers of macrophages, monocytes, and lymphocytes (Figure 3E). Both frequency and absolute numbers of neutrophils were not modified by Ang-(1-7) (Figure 3, D and E).

**Figure 4:**
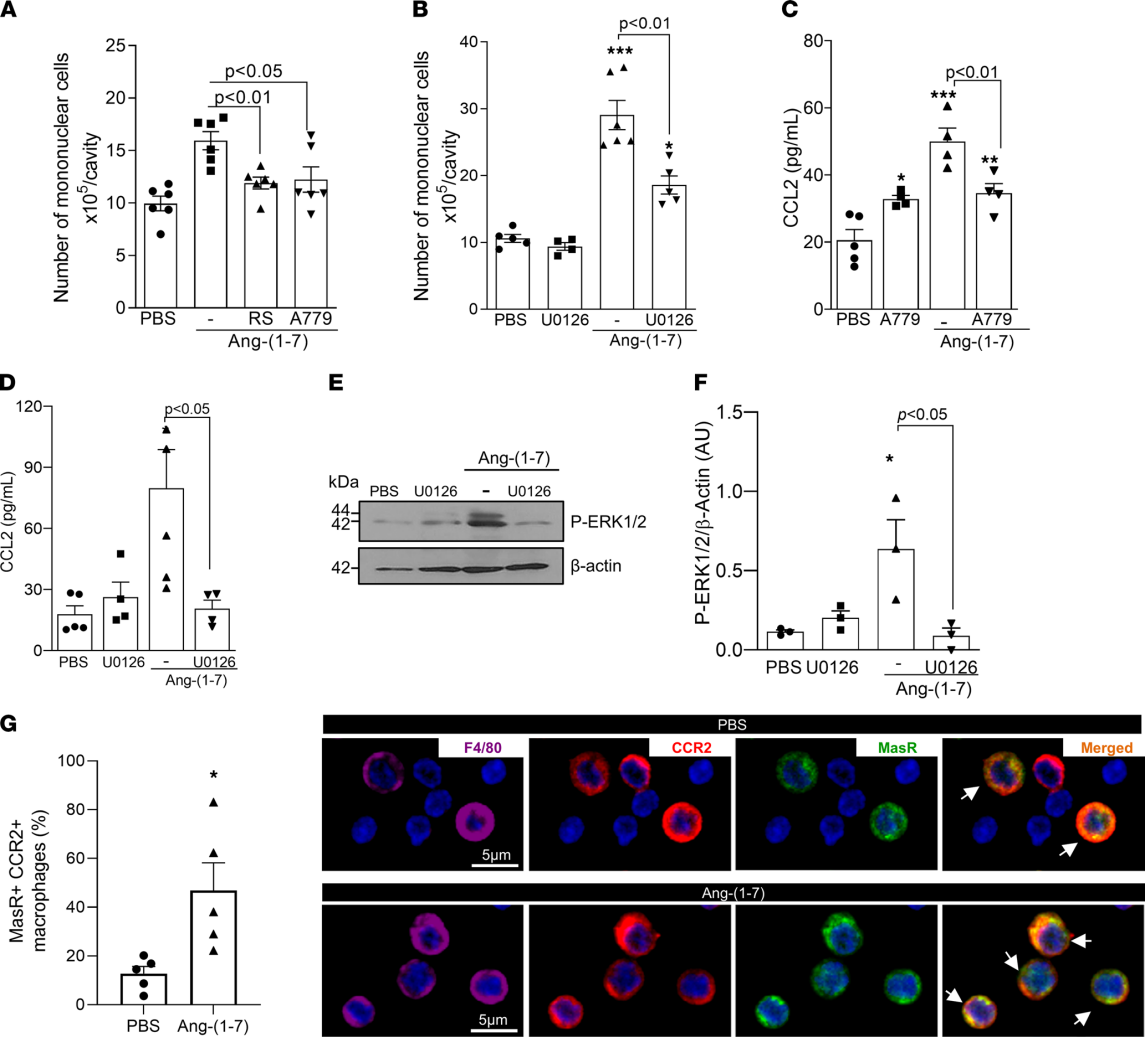
MasR, CCR2, and the MEK/ERK1/2 pathway are important for Ang-(1-7)–mediated recruitment of macrophages. BALB/c mice were treated with RS504393 (2 mg/kg i.pl.), A779 (200 ng/cavity), or U0126 (60 μg/cavity) 1 hour before the local injection of Ang-(1-7) (100 ng/cavity). Leukocyte recruitment to the pleura cavity was evaluated at 48 hours post–Ang-(1-7) (**A** and **B**). CCL2 levels were measured at 6 hours post–Ang-(1-7) in the cell-free pleural lavage supernatants (**C** and **D**). Phosphorylation of ERK1/2 was evaluated by Western blotting of recruited leukocytes at 48 hours post–Ang-(1-7) (**E** and **F**). At the same time point, frequencies of CCR2^+^MasR^+^ macrophages were determined by confocal microscopy (**G** — arrows indicate the double-positive cells). Results are shown as the mean ± SEM of *n* = 4-6 mice in each group. * for *P* < 0.05, ** for *P* < 0.01, and *** for *P* < 0.001 when compared with the control group (PBS) by 1-way ANOVA (**A**–**F**) or *t* test (**G**).

Akin to our in vitro data, pretreatment of mice with RS504393, or A779, decreased Ang-(1-7)–induced recruitment of mononuclear cells into the pleural cavity (Figure 4A). Given the critical role of the MEK/ERK pathway in the induction of CCL2-mediated macrophage migration (20, 22) and that Ang-(1-7) induced a time-dependent increase in ERK1/2 phosphorylation in leukocytes recruited to the pleura (Supplemental Figure 1, C and D), we treated mice with the MEK/ERK inhibitor U0126, 1 hour before Ang-(1-7) injection. Inhibition of the MEK/ERK pathway significantly reduced the recruitment of mononuclear cells observed at 48 hours post–Ang-(1-7) (Figure 4B). Of note, the Ang-(1-7)–mediated increase in CCL2 was also reduced by the inhibition of the MasR and MEK/ERK pathway (Figure 4, C and D). As expected, the pharmacological inhibition of MEK/ERK decreased the Ang-(1-7)–induced phosphorylation of ERK1/2 (Figure 4, E and F). Next, confocal microscopy experiments were performed to determine whether CCR2 receptor colocalized with MasR in Ang-(1-7)–recruited macrophages. Interestingly, an increased frequency of macrophages with concomitant expression of CCR2 and MasR was observed in the pleural cavity of mice 48 hours post–Ang-(1-7) injection (Figure 4G — arrows indicate the MasR^+^ CCR2^+^ cells). Together, these data provide evidence that Ang-(1-7), acting through MasR, activates the MEK/ERK pathway, leading to the production of CCL2 and recruitment of mononuclear cells via CCR2.

### Macrophages recruited by Ang-(1-7) present regulatory phenotypes.

**Figure 5:**
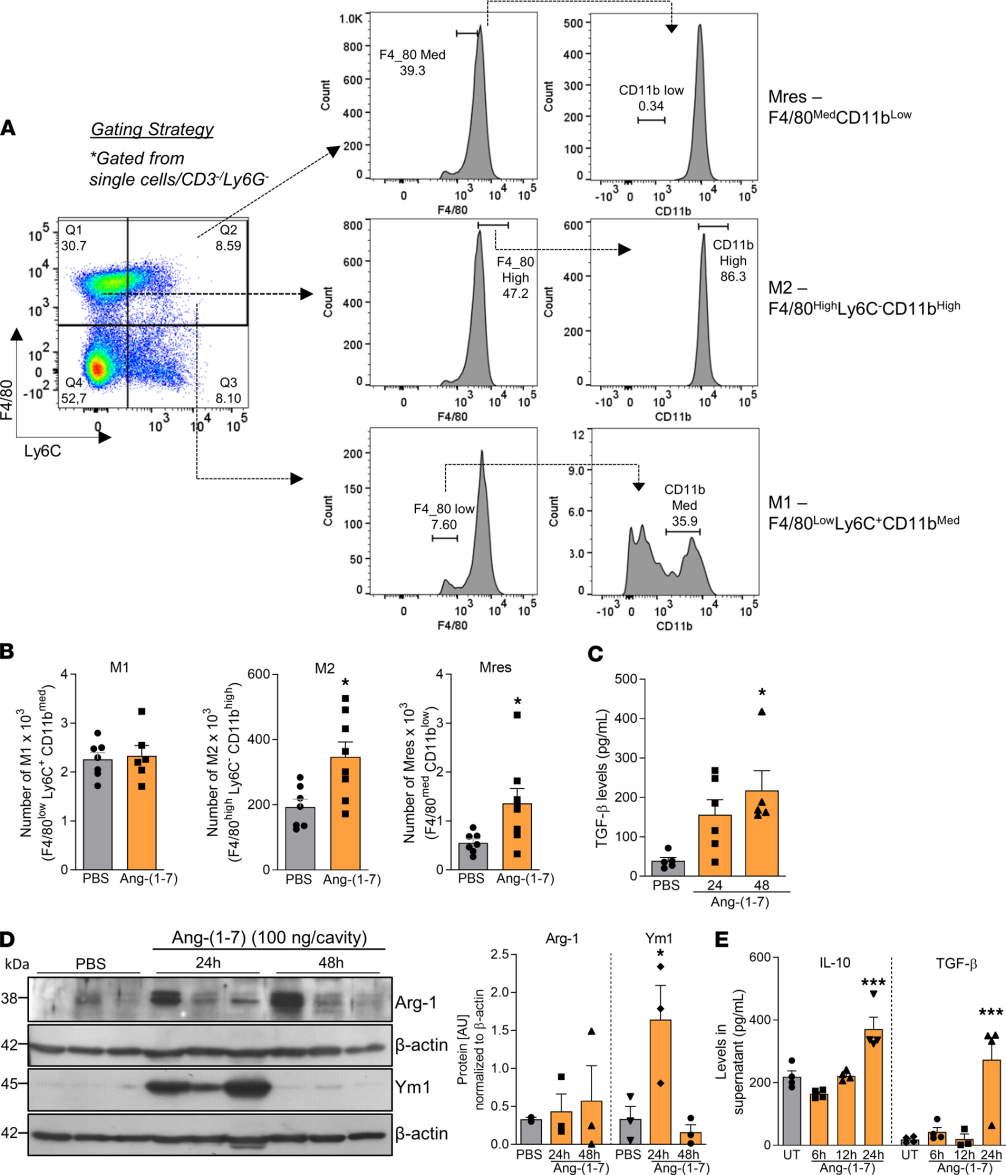
Macrophages recruited to the pleura post–Ang-(1-7) injection present a regulatory phenotype. Briefly, BALB/c mice received an i.pl. injection of Ang-(1-7) (100 ng/cavity) or PBS (controls), and the macrophages recruited to the cavity were harvested at 48 hours for phenotyping by flow cytometry as shown in gating strategy (**A**). (**B**) Graphs present the absolute numbers of M1 (F4/80^lo^Ly6C^+^CD11b^med^), M2 (F4/80^hi^Ly6C^–^CD11b^hi^), and Mres (F4/80^med^CD11b^lo^) recruited into the pleura. TGF-β levels were assessed in the pleural lavage supernatant from Ang-(1-7)–injected mice at different time points postinjection (**C**). Leukocytes recruited into the pleural cavity were processed for Western blot analysis of Arg-1 and Ym1 levels (**D**). β-Actin was used as a loading control. During in vitro settings, the kinetics of production of IL-10 and TGF-β by BMDMs were evaluated (**E**). Data are presented as mean ± SEM of 8 mice per group (in vivo) or are representative results of 3 independent experiments with BMDMs performed in biological quadruplicates (*n* = 4). Western blot quantification was performed using ImageJ software from the representative blots shown in **D**, which used whole cell extracts from 3 mice. * for *P* < 0.05 and *** for *P* < 0.001 when compared with the control group (PBS) by *t* test (**B**) or 1-way ANOVA (**C** and **E**).

Macrophage phenotype was evaluated based on 3 populations previously identified (23–25) (Figure 5A). Ang-(1-7) injection increased the numbers of M2 (F4/80^hi^Ly6C^–^CD11b^hi^) and proresolving macrophages (Mres — F4/80^med^CD11b^lo^) in the pleura of mice while numbers of classically activated (M1 — F4/80^lo^Ly6C^+^CD11b^med^) remained unmodified (Figure 5B). The regulatory phenotype of macrophages was consistent with the increased production of IL-10 (Figure 3B) and TGF-β (Figure 5C) observed post–Ang-(1-7) injection. Besides, Arginase 1 (Arg1) and Ym1, classical M2 markers, were increased in Ang-(1-7)–recruited leukocytes (Figure 5D). Of note, Ang-(1-7) also increased BMDMs’ production of IL-10 and TGF-β in a time-dependent manner (Figure 5E).

Corroborating our in vivo data, Ang-(1-7) preferentially induced migration of M2-like macrophages (IL-4 polarized) while preventing migration of M1 cells (LPS+IFN-γ polarized) (Supplemental Figure 3).

### MasR is crucial for the recruitment of mononuclear cells during resolution of inflammation.

**Figure 6:**
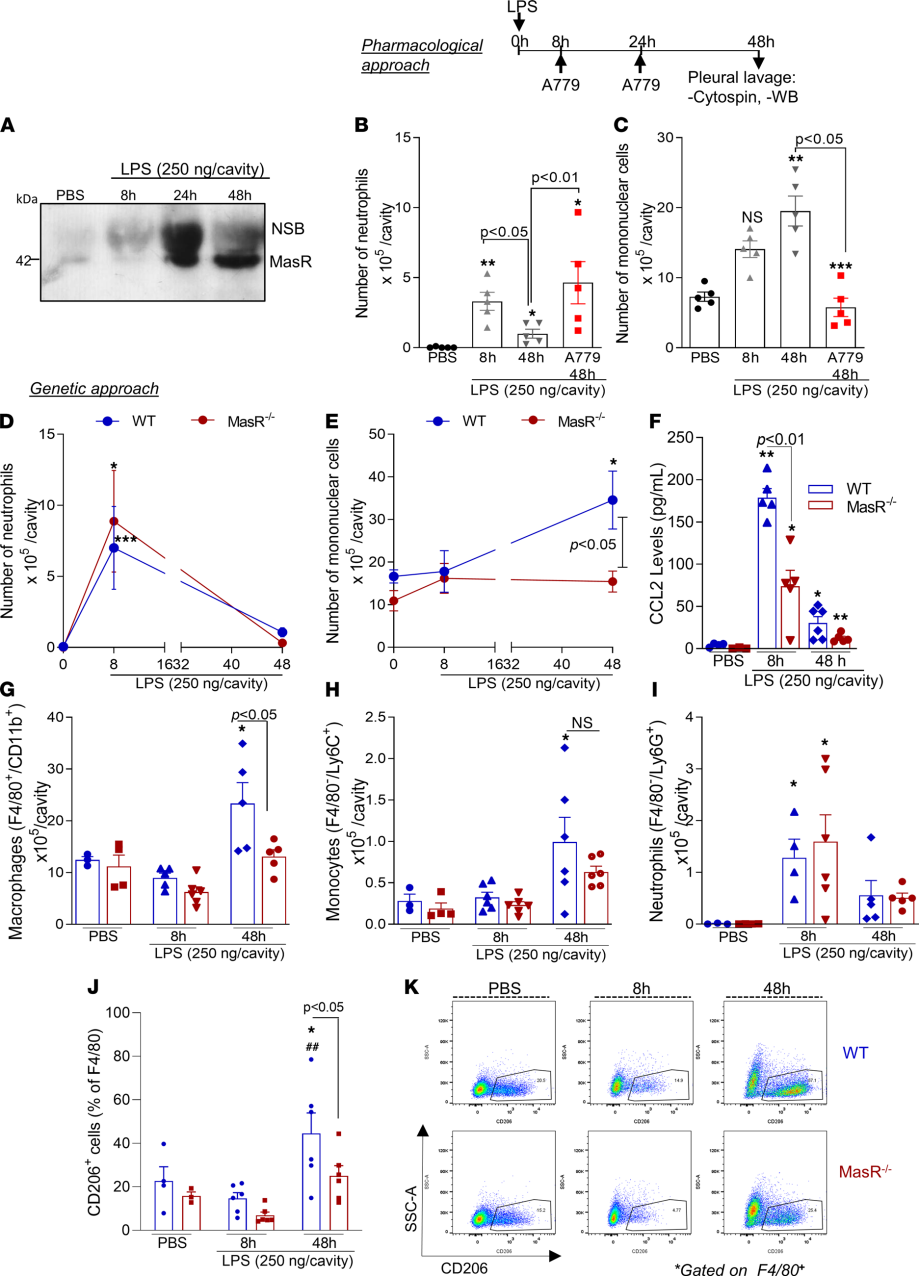
MasR is upregulated during the resolution of inflammation and is important for recruitment of regulatory macrophages. BALB/c mice were challenged with LPS (250 ng/cavity, i.pl.) or PBS, and leukocytes from the pleural cavity were harvested after 8, 24, and 48 hours for Western blot analysis of MasR (**A**) and differential cell counts (**B** and **C**). LPS-challenged mice were treated with A779 (200 ng/cavity) or vehicle at 8 and 24 hours post-LPS injection, and leukocytes were harvested at 48 hours for differential cell counts (**B** and **C**). Next, WT and MasR^–/–^ mice were also i.pl. challenged with LPS, and neutrophil (**D**) and mononuclear cell numbers (**E**) and CCL2 levels (**F**) were evaluated. Flow cytometry analysis was performed to assess numbers of macrophages (F4/80^+^CD11b^+^ — **G**), monocytes (F4/80^–^Ly6C^+^ — **H**), and neutrophils (F4/80^–^Ly6G^+^ — **I**). Frequencies of CD206^+^ macrophages are graphed in **J** and representative gating is shown in **K**. Results are shown as the mean ± SEM of *n* = 5–6 mice. * for *P* < 0.05, ** for *P* < 0.01, and *** for *P* < 0.001 when compared with the control group (PBS). ^##^ for *P* < 0.01 when compared with the 8-hour time point, or as indicated, by 1-way ANOVA (**B** and **C**) or 2-way ANOVA (**D**–**K**). NSB, nonspecific band.

The relevance of endogenous Ang-(1-7)–induced migration of mononuclear cells was evaluated in a well-characterized self-resolving model of LPS-induced pleurisy (26–28). In this model, the intrapleural injection of LPS promotes early recruitment of neutrophils to the pleural cavity that peaks at 8 hours postchallenge, decreasing thereafter. At the resolution phase (48 hours post-LPS), a significant recruitment of mononuclear cells was observed concomitant with the decline in neutrophils’ numbers (Figure 6, B and C). Notably, MasR expression in the pleural recruited leukocytes was significantly upregulated at this time point (Figure 6A).

Local administration of A779 at the peak of inflammation prevented the spontaneous reduction of neutrophil numbers (Figure 6B) and the increase of mononuclear cell recruitment (Figure 6C) seen at 48 hours in the vehicle group. These data reinforce that the endogenous Ang-(1-7)/MasR axis is indeed part of a proresolutive physiological program.

To further evaluate the role of MasR in the resolution of inflammation, we compared the kinetics of leukocyte infiltration after LPS challenge in MasR^–/–^ and WT controls. Although the genetic deficiency in MasR did not modify neutrophil infiltration post-LPS in the evaluated time points (Figure 6D), the number of mononuclear cells recruited to the pleural cavity was significantly lower in MasR^–/–^ compared with WT mice (Figure 6E). Accordingly, pleural levels of the chemokine CCL2 were significantly lower in MasR^–/–^ when compared with WT mice (Figure 6F). Flow cytometry (gating strategy — Supplemental Figure 4) showed lower numbers of macrophages, but not monocytes and neutrophils, in MasR^–/–^ in comparison with WT (Figure 6, G–I). Of interest, the frequency of CD206-positive macrophages, an M2 marker necessary for the engulfment of apoptotic bodies (29), was strikingly lower in the pleural exudate of MasR^–/–^ mice (Figure 6, J and K), indicating that MasR might affect macrophage mechanisms for efferocytosis. To validate our findings, we tested whether the absence of MasR would influence the neutrophil clearance in a more severe model of inflammation. During severe pleurisy, MasR^–/–^ mice presented higher numbers of neutrophils during early stages (i.e., 8 hours postchallenge) and at the resolution phase of inflammation (48 hours postchallenge) compared with WT animals (Supplemental Figure 5A). Furthermore, we observed a decreased infiltration of macrophages at 8 and 24 hours post-LPS and a rebound of recruitment at 48 hours (Supplemental Figure 5B). Although macrophages did arrive late in MasR^–/–^ mice, they could not promote neutrophil removal from the pleural cavity at the 48-hour time point. This was accompanied by a lower frequency of efferocytic events in the MasR^–/–^ animals (Supplemental Figure 5C).

In sum, pharmacological and genetic inhibition of MasR uncovered its essential role in maintaining mononuclear cell migration, a key step for an effective resolution of inflammation.

### Ang-(1-7)/MasR axis promotes production of CCL2, recruitment of macrophages, and phagocytosis of bacteria in a self-resolving model of peritonitis.

**Figure 7:**
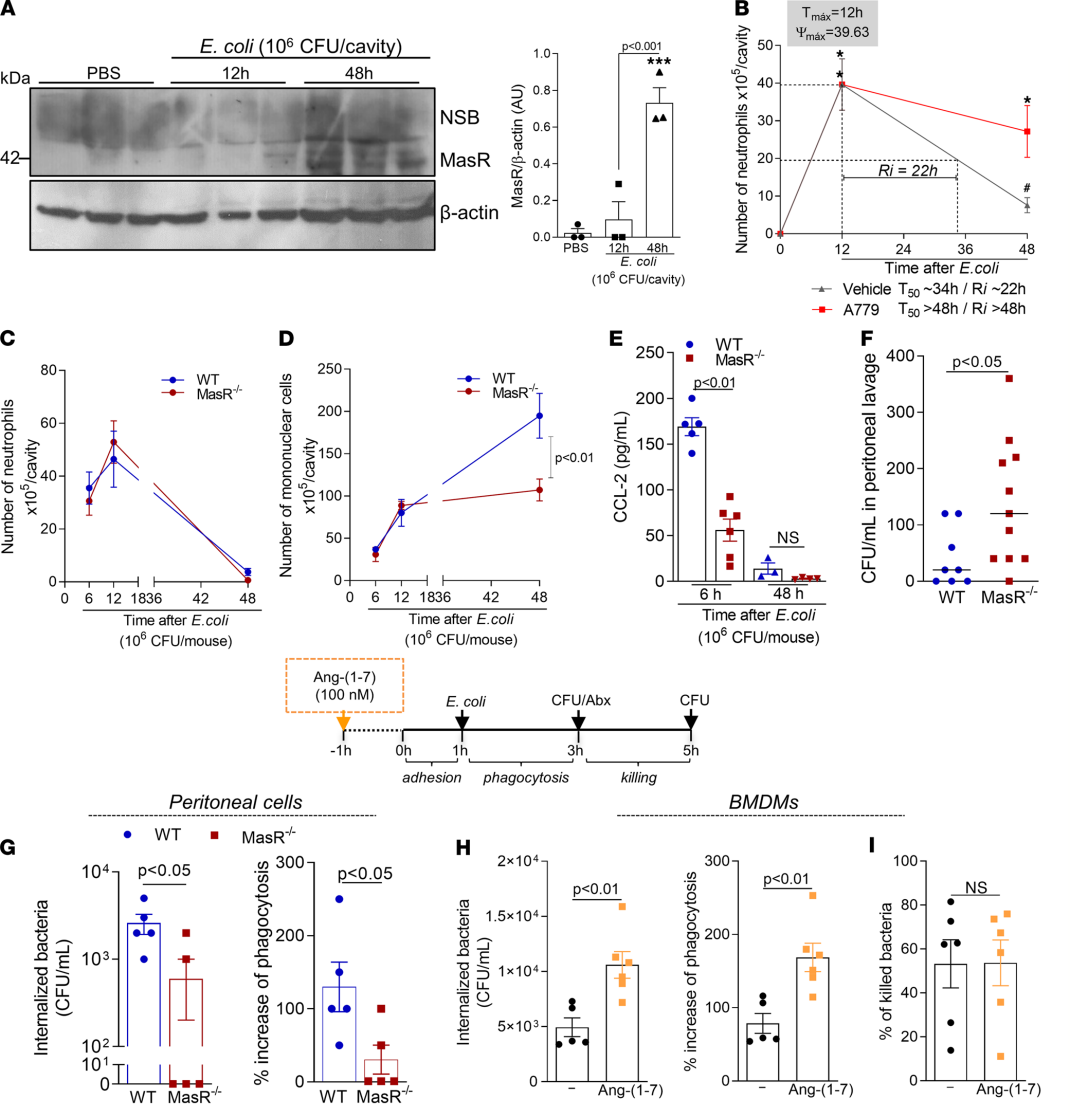
The Ang-(1-7)/MasR pathway promotes the recruitment of macrophages, production of CCL2, and phagocytosis of bacteria. WT mice were infected with *E*. *coli* (1 × 10^6^ CFU), and macrophages were harvested at 12 and 48 hours postinfection for MasR expression (**A**, *n* = 3). Macrophages from PBS-injected mice were used as controls. Next, infected mice were treated with A779 (200 ng/cavity) or vehicle, and neutrophil numbers at 12 and 48 hours postinfection were evaluated to calculate the resolution intervals (Ri — **B**). T50, time point when neutrophil numbers reduced to 50% of maximum (*n* = 7). In addition, WT and MasR^–/–^ mice were infected, and the numbers of neutrophils (**C**) and macrophages (**D**) was evaluated at different time points postinfection. CCL2 levels were measured in the cell-free supernatants of the peritoneal lavages at 6 and 48 hours postinfection (**E**). (**F**) Graph shows the CFU numbers in the lavage at 6 hours postinfection (*n* = 5–11). Phagocytosis of bacteria was evaluated in peritoneal macrophages from naive WT and MasR^–/–^ (**G**) or WT BMDMs pretreated with Ang-(1-7) — 100 nM (**H**). Results are expressed as CFU of internalized bacteria or percentage of phagocytosis (*n* = 5–6). In a parallel experiment, macrophages were incubated for another 2 hours after antibiotics to assess the killing of bacteria inside the macrophages by evaluating the number of viable bacteria (CFU counts in LB agar plates — **I**). Data are presented as the mean ± SEM, * for *P* < 0.05 and *** for *P* < 0.001, when compared with the control group (PBS), or ^#^ for *P* < 0.05 when compared with the A779-treated group, by 1-way ANOVA or *t* test (when comparing 2 groups).

To evaluate the role of MasR in infections, a self-resolving model of peritonitis was performed (30). Macrophage expression of MasR increased at the resolution phase (48 hours) but not at the peak (12 hours) of *E*. *coli*–elicited peritonitis (Figure 7A). Importantly, blockage of MasR at the peak of inflammation significantly delayed resolution of neutrophilic inflammation during *E*. *coli* peritonitis, as evidenced by the increased resolution interval observed (Ri = 22 hours in the vehicle group vs. Ri > 48 hours in the A779-treated group — Figure 7B).

Next, WT and MasR^–/–^ mice were infected intraperitoneally with *E*. *coli* and evaluated after different time points postinfection. In agreement with data from the pleurisy model, deficiency of MasR impaired the recruitment of macrophages during the resolution phase of *E*. *coli*–induced peritonitis, without altering neutrophil kinetics of recruitment into the peritoneal cavity (Figure 7, C and D). The reduced numbers of macrophages were associated with a significant reduction of CCL2 levels during infection (Figure 7E) and higher bacteria counts in the MasR^–/–^ mice in comparison with WT (Figure 7F).

To test whether Ang-(1-7)/MasR would impact macrophage ability to phagocytose bacteria, peritoneal macrophages from naive WT or MasR^–/–^ were isolated, and 2 × 10^5^ cells from each group were plated for an in vitro phagocytosis assay (see Methods). Phagocytosis of *E*. *coli* was impaired by MasR deficiency (Figure 7G) and enhanced when BMDMs of WT mice were treated in vitro with Ang-(1-7) (Figure 7H). Besides the increased phagocytosis, Ang-(1-7) treatment did not enhance macrophage-related bacterial killing (Figure 7I). Of interest, Ang-(1-7) did not display direct classic antibacterial activity (Supplemental Figure 6). Altogether, Ang-(1-7):MasR is important for macrophage phagocytosis of bacteria, but it does not seem to impact the mechanisms for pathogen killing in macrophages. Thus, the higher bacteria loads observed in vivo in MasR^–/–^ mice compared with WT (evidenced in Figure 7F) are probably due to the decreased uptake of bacteria that, over time, leads to an overall delayed *E*. *coli* clearance.

### Ang-(1-7)/MasR axis is a regulator of efferocytosis in vivo and in vitro.

Efferocytosis is a critical event in the resolution of inflammation (31). Previously, we have shown that Ang-(1-7) promotes efferocytosis of apoptotic neutrophils (11) and eosinophils (13); yet, the associated mechanisms remained unknown.

**Figure 8:**
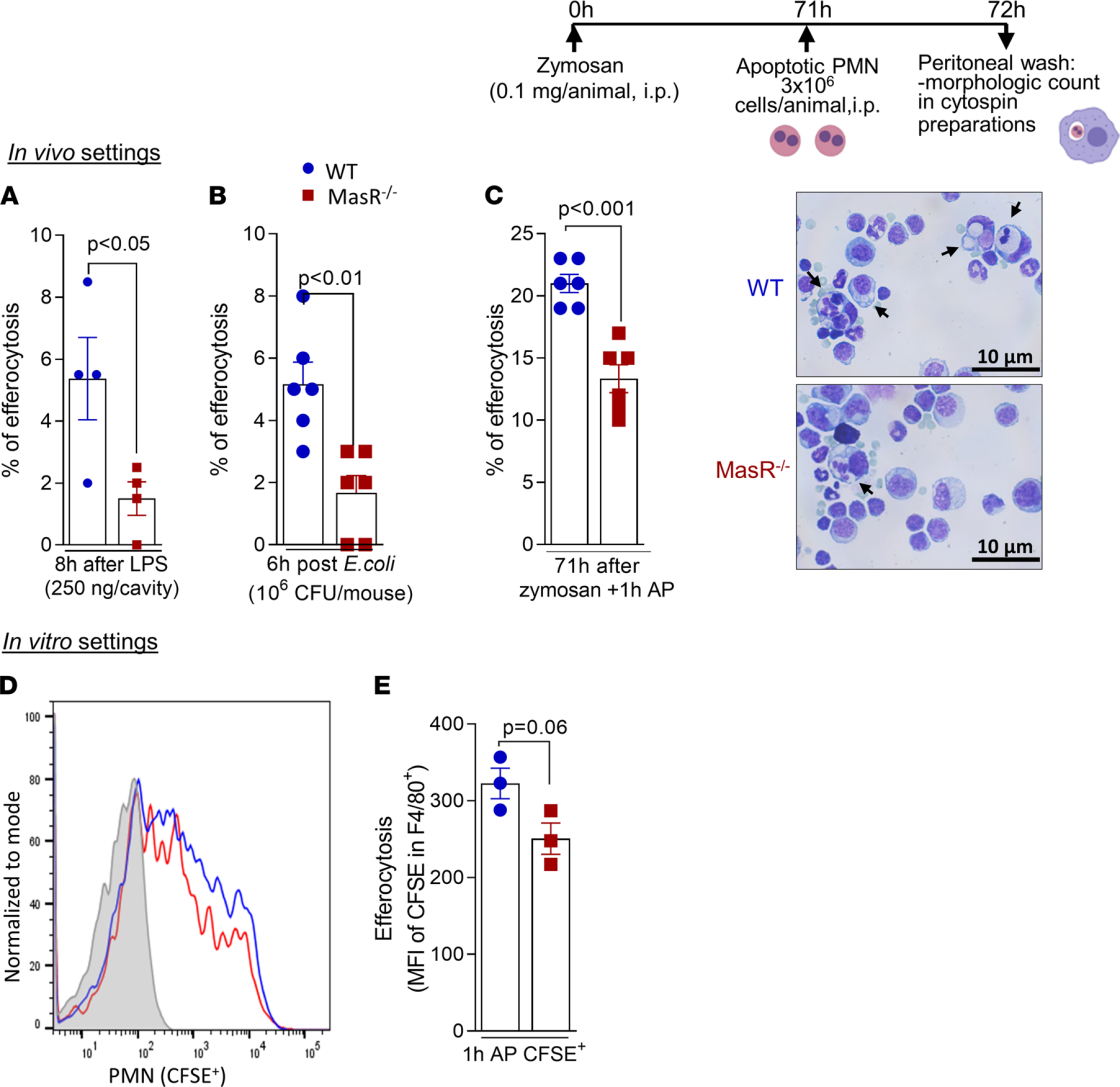
MasR is important for efferocytosis of apoptotic neutrophils. WT and MasR^–/–^ mice received an i.pl. injection of LPS (250 ng/cavity) (**A**) or were infected with *E*. *coli* intraperitoneally (**B**), and efferocytosis was morphologically identified in cytospin slides at 8 and 6 hours postchallenge, respectively. The frequency of efferocytosis was evaluated by counting 500 cells per slide. WT and MasR^–/–^ were also used for the efferocytosis assay post-zymosan intraperitoneal injection, as shown in experimental design above the figure. Percentage of efferocytosis was obtained by morphological identification in cytospin slides (**C**). Representative images of the slides are shown in **C**. Original magnification, 100×. Arrows represent macrophages with engulfed apoptotic cells. Data are shown as the mean ± SEM of *n* = 4–6 mice in each group. Lastly, CFSE-labeled neutrophils were coincubated with WT or MasR^–/–^ BMDMs for 1 hour, and flow cytometry was performed for efferocytosis evaluation (MFI of CFSE in macrophages — **D** and **E**). PMN, polymorphonuclear cell.

Because the frequency of CD206 was reduced in macrophages from MasR^–/–^ mice post-LPS challenge (Figure 6, J and K), we initially analyzed efferocytosis during this self-resolving model of pleurisy. In WT mice, the rate of neutrophil efferocytosis reflected the pattern of neutrophilic infiltration, being maximal at the peak of inflammation (8 hours) and decreasing thereafter (Supplemental Figure 7A). Although efferocytosis was relatively low at 48 hours post-LPS, the antagonism of MasR induced a further decrease in the frequency of efferocytosis (Supplemental Figure 7B). In addition, a lower frequency of macrophage efferocytosis was observed in MasR^–/–^ mice in comparison with WT mice during both models of self-resolving inflammation: LPS-induced pleurisy and *E*. *coli* peritonitis (Figure 8, A and B).

To validate our findings, we next performed a well-known efferocytosis assay by injecting prey apoptotic neutrophils into the peritoneal cavity of WT and MasR^–/–^ mice bearing 71-hour peritonitis elicited by zymosan (24, 25, 32). Once again, the engulfment of apoptotic neutrophils by MasR^–/–^ macrophages was lower when compared with WT cells (Figure 8C and representative slide images).

**Figure 9:**
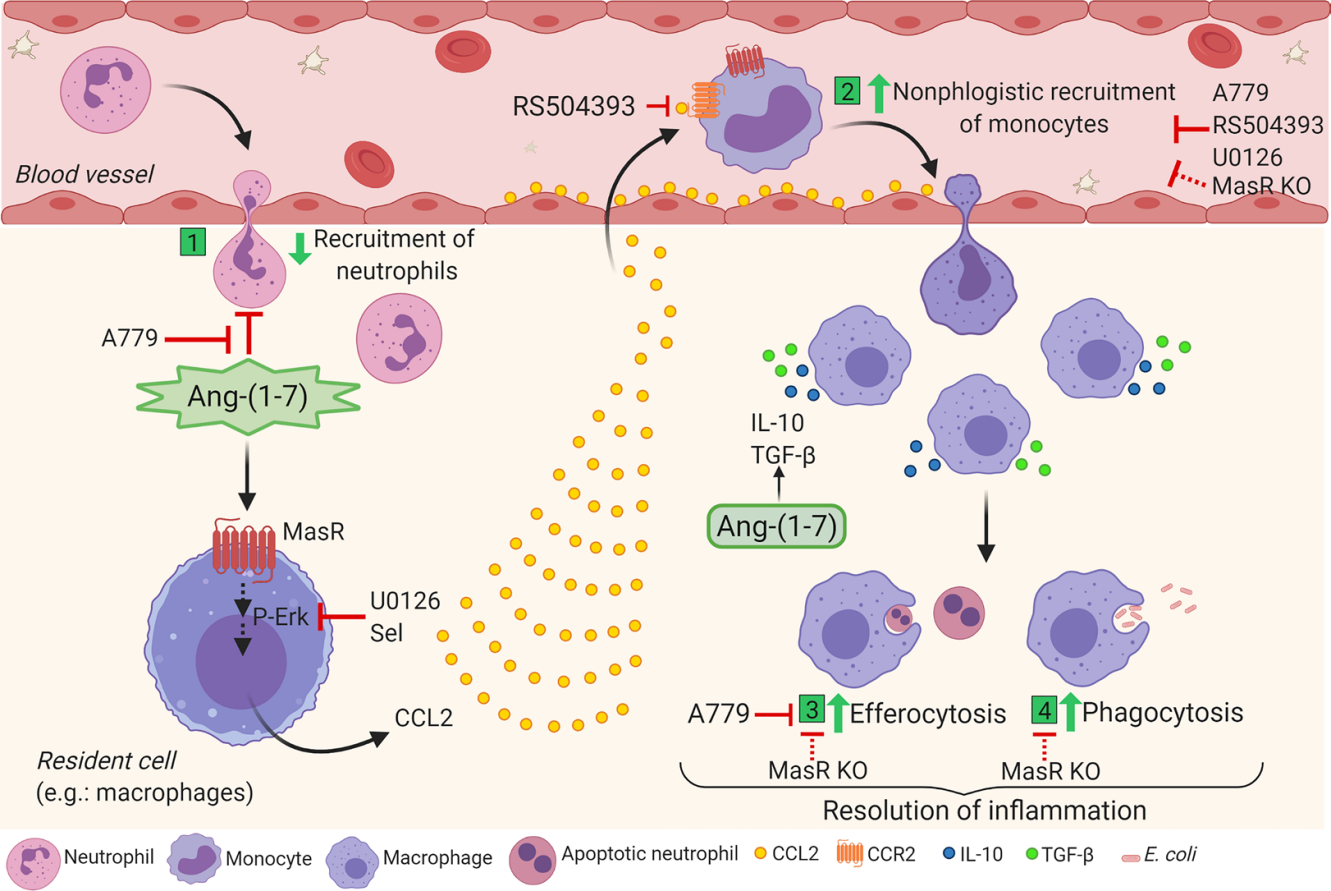
Schematic representation of Ang-(1-7) mechanisms in macrophage migration and function favoring resolution of inflammation. (1) Ang-(1-7) impairs the migration of neutrophils in a MasR-dependent way. (2) On the other hand, Ang-(1-7) signals through its MasR, culminating in the phosphorylation of ERK1/2 and production of CCL2, probably by resident cells. CCL2 binds to CCR2 receptor and promotes nonphlogistic monocyte migration, which can be abrogated by the blockage of CCR2 (RS504393), antagonism or absence of MasR (A779 and Mas knockout) or inhibition of ERK signaling (U0126). In addition, Ang-(1-7)/MasR takes part in the spontaneous resolution of acute inflammation induced by LPS or *E*. *coli* by promoting nonphlogistic migration of regulatory monocytes/macrophages and engaging in the removal of apoptotic neutrophils through efferocytosis (3) and bacteria through phagocytosis (4) and production of IL-10 and TGF-β. The proresolving effects summarized herein account for the endogenous role of Ang-(1-7) in the physiological resolution of inflammation. Note: red lines represent inhibitory effects. Created with BioRender.

In agreement with our in vivo data, we observed a trend toward lower rates of efferocytosis in BMDMs from MasR^–/–^ (MFI of CFSE in F4/80^+^) in comparison with WT cells (Figure 8, D and E). The results gathered here provide mechanistic evidence that Ang-(1-7)/MasR enhances macrophage responses that are important for the resolution of inflammation, including recruitment of regulatory cells, phagocytosis of bacteria, efferocytosis, and production of regulatory cytokines (Figure 9).

## Discussion

Resolution of inflammation is a time-regulated process that results in the termination of the inflammatory response to restore tissue homeostasis (33). Endogenous proresolving mediators orchestrate resolution by promoting apoptosis and efferocytosis of granulocytes, reducing the levels of proinflammatory mediators, and inducing the nonphlogistic recruitment and polarization of macrophages toward regulatory phenotypes (34). Understanding the mechanisms of action of proresolving molecules aids the development of new therapeutic opportunities for inflammatory diseases (35).

Ang-(1-7), initially identified as an inactive metabolite of Ang II (36), was recently shown to induce features of resolution of inflammation (11–13). Still, the mechanisms involved are poorly explored. Here, we have identified a potentially new proresolving feature of Ang-(1-7), namely the nonphlogistic recruitment of monocytes/macrophages. In summary, we have shown that Ang-(1-7) (i) induces migration of murine and human macrophages but not neutrophils, (ii) promotes CCL2/CCR2–dependent recruitment of mononuclear cells to the pleural cavity associated with secretion of the regulatory cytokines IL-10 and TGF-β, (iii) induces expression of M2-like markers in recruited macrophages, and (iv) is important for the clearance of bacteria and apoptotic cells promoting phagocytosis and efferocytosis, all critical features of the resolution of inflammation. In addition, (v) macrophages recruited by Ang-(1-7) presented concomitant expression of CCR2 and MasR, indicating that both pathways might be active in the same cell. Mechanistically, the Ang-(1-7)–mediated recruitment of macrophages was (vi) dependent on MasR and (vii) dependent on the activation of the MEK/ERK pathway upstream to CCL2 and CCR2.

Macrophages are extremely plastic cells that polarize to perform specific tasks depending on the cues from the tissue milieu — from inflammation induction to resolution (37). The contrasting functions of macrophages are related to the different cell phenotypes characterized by distinct expression of surface markers, metabolism programs, and production of mediators (15, 37, 38). Macrophages recruited during the early steps of inflammation are activated and secrete proinflammatory cytokines and other mediators, orchestrating the inflammatory process (37, 39). In contrast, once the inflammatory stimuli are neutralized/cleared, a shift in the production from inflammatory proresolving mediators takes place in the tissue (14). In addition to the reprogramming to a counterregulatory phenotype, significantly increased numbers of macrophages are usually observed in the tissue during resolution phase of inflammation (16). The proresolving macrophages are particularly important for the phagocytosis of bacteria/debris, for the efferocytosis of apoptotic granulocytes, and to mediate tissue regenerative responses (40). Traditionally, macrophages are artificially divided into M1 and M2 phenotypes, also known as classically or alternatively activated macrophages, respectively. In vivo, a distinct population of macrophages that appears in the inflammatory site during the resolution phase of inflammation was also identified and named Mres or proresolving macrophages (41). During resolution, M2 and Mres macrophages are important players (23, 42). Of interest, M2 macrophages are highly efferocytic and secrete antiinflammatory cytokines, including IL-10 and TGF-β (43, 44). The differences in macrophage responses/profiles are also noticed in the distinct mouse strains used for experimental research. For instance, BALB/c and C57BL/6 macrophages present a distinct magnitude of inflammatory responses to LPS (45). Nevertheless, here we observed a very similar migration response in both BALB/c and C57BL/6 mice and a comparable pattern in the self-resolving model used for our experiments, minimizing the possibility of a strain-specific effect in the immunological response observed (46).

CCL2 is a canonical macrophage chemoattractant. Increased CCL2 in MasR-deficient mice upon inflammatory stimulation or in the context of experimental autoimmune encephalomyelitis (EAE) was shown to promote the recruitment of inflammatory macrophages (9). On the other hand, CCL2 has been recognized as a crucial cytokine for the recruitment of monocytes and induction of M2/Mres macrophages’ phenotypes (16, 47). Indeed, CCL2 was shown to induce polarization of human and murine macrophages to an M2 phenotype acting via CCR2 (48, 49) and enhances apoptotic cell removal by macrophages, activating this important proresolving cellular function (50). Proresolving agents such as cAMP and plasmin (16, 20, 24) mediate the nonphlogistic recruitment of macrophages in a CCR2:CCL2–dependent way. Therefore, given the continuum of phenotypes that macrophages present and that these cells can be activated by different environment cues, the role of CCL2:CCR2 in the polarization of macrophages to a specific phenotype is context dependent. Here, Ang-(1-7), acting through MasR, promoted a rapid secretion of CCL2 — as early as 4 hours after Ang-(1-7) exposure in vitro and 6 hours after in vivo injection — by monocytes/macrophages, enhanced the noninflammatory monocyte recruitment via CCR2, and increased efferocytosis, in agreement with previous reports (11). However, whether Ang-(1-7) directly or indirectly binds to CCR2 to induce chemotaxis is yet to be fully explored. In contrast, migration and activation of macrophages are inhibited by Ang-(1-7) in inflammatory conditions (ref. 18; Figure 1, E and F; and Supplemental Figure 3). Thus, the state of activation of cells and previous exposure to inflammatory stimuli determine Ang-(1-7) actions on macrophages. Several studies concur with ours in that Ang-(1-7) can deactivate proinflammatory macrophages (12, 17, 18, 51, 52), which could explain the decreased migration toward an inflammatory stimulus observed in our results. Importantly, an increased frequency of MasR^+^CCR2^+^ macrophages was detected in the pleura of mice at 48 hours post–Ang-(1-7) injection. Prior reports show that MasR can modulate the activity of other receptors, such as AT1 and AT2, by hetero-oligomerization (53, 54). Although a direct interaction between MasR and CCR2 has not yet been described, here, we show that macrophages recruited post–Ang-(1-7) harbor both receptors, suggesting that the 2 pathways are active in the same cell and are contributing to the outcome observed: recruitment of monocytes that further turn into macrophages with proresolving actions. Numerous reports in the literature have already shown cooperation between receptors, leading to efficient chemotaxis of leukocytes. Indeed, a receptor for a given mediator can be critical for the chemotaxis of leukocytes toward a different mediator (that also binds to another receptor) (55–57). Here, we used suboptimal concentrations of CCL2 and observed that while CCL2 induces a significant increase in WT BMDMs’ migration, BMDMs from MasR^–/–^ do not migrate toward CCL2. Therefore, our data suggest that Ang-(1-7) leads to CCL2 production and that CCR2 and MasR seem to act in cooperation, leading to macrophage migration toward Ang-(1-7) in the presence of CCL2. Additional studies will further elucidate the complete mechanisms by which MasR and CCR2 cooperate to induce migration.

Cellular migration requires specific intracellular signaling events, including the MEK/ERK pathway (20, 58–60). Here, this signaling pathway was critical for Ang-(1-7) actions as confirmed by in vitro and in vivo experiments. Ang-(1-7), most likely through MasR binding, induced the activation of the MEK/ERK pathway, which was associated with the production of CCL2 and recruitment of monocytes/macrophages. Our findings are in accordance with previous studies that observed MasR-dependent increased ERK1/2 phosphorylation after Ang-(1-7) (61). Of note, the ERK1/2 pathway has been suggested to promote regulatory actions in macrophages, enhancing the production of IL-10 by these cells (62).

In vitro and in vivo recruitment of monocytes/macrophages induced by Ang-(1-7) was accompanied by increased secretion of IL-10 and TGF-β, rather than the production of proinflammatory mediators (nonphlogistic). Akin with our results, IL-10 production was previously triggered by the agonism of MasR with Ang-(1-7) or derived peptides (17, 63). Also, the proresolving mediator lipoxin A_4_ was shown to increase the production of IL-10 via the Ang-(1-7)/MasR axis in an experimental model of acute lung injury (64). Given the importance of IL-10 and TGF-β as markers of M2-like macrophages (65) and further induction of efferocytosis (66–69), we can suggest that induction of these regulatory cytokines by Ang-(1-7) might contribute to the promotion of efferocytosis and polarization to regulatory phenotypes of macrophages found in the tissue. Indeed, IL-10–producing macrophages are highly efferocytic (66), and Ang-(1-7)–recruited macrophages presented high expression of the classical M2 markers Arg1 and Ym1. Further evaluation of the cellular sources of IL-10 and TGF-β after Ang-(1-7) injection will more comprehensively uncover all the players involved in the induction of Ang-(1-7) antiinflammatory/proresolving actions.

In keeping with our results, BMDMs from MasR^–/–^ mice presented decreased expression of M2 markers in M2-differentiated (IL-4) macrophages, but increased expression of M1-related (IFN-γ/LPS) genes (9). In the same vein, we have previously found decreased expression of activation markers after exposure of M1 (IFN-γ/LPS) macrophages to Ang-(1-7), shifting the phenotype toward regulatory cells in vitro (12). Moreover, Ang-(1-7) treatment of LPS-inflamed mice promotes resolution of inflammation associated with decreased frequency of M1 macrophages into the pleural space (12). Of interest, we have observed a low-range expression of inducible NOS in pleural cells post–Ang-(1-7) injection into the pleura (data not shown). Given that Ang-(1-7)/MasR increased the phagocytic activity of macrophages to bacteria, we hypothesize that this mixed phenotype of macrophages induced by Ang-(1-7) preserves the macrophage antimicrobial responses while preventing exacerbations of inflammation. Indeed, previous studies have observed that harnessing this alternative branch of the RAS using agonists of MasR favors the phagocytic activity of dysfunctional neutrophils of diabetic animals (70). Our self-resolving model of *E*. *coli–*induced peritonitis replicated the findings from the LPS-induced pleurisy, supporting the important role of MasR inducing the CCL2-mediated migration of macrophages during the resolution phase of infection and clarifying its importance for macrophage phagocytosis of bacteria by aiding the resolution of infections. Recently, we have also shown that Ang-(1-7) decreases viral burden in the lungs during influenza A infection, without acting directly on the viral killing/inhibition (71). Here, in agreement with this previous study, we have shown that Ang-(1-7) did not present a direct antimicrobial effect to *E*. *coli*. Therefore, the protective effects of Ang-(1-7) are due to the modulation of the innate immune cells. In summary, despite inducing recruitment of monocytes/macrophages at steady-state conditions or during the resolution phase of inflammation, Ang-(1-7) significantly reduces recruitment of neutrophils and inflammatory macrophages if administered to inflamed mice (10, 12, 52), while enhancing the antimicrobial potential of macrophages (72).

Neutrophils can be directly affected by Ang-(1-7) given MasR expression in these cells (11). Ang-(1-7) exposure to murine and human neutrophils significantly deactivates proinflammatory pathways of these cells (11). In addition, harnessing the MasR pathway was shown to reduce leukocyte rolling and adhesion in a model of arthritis, leading to reduced recruitment of neutrophils in the joint (10). In line with this, MasR^–/–^ mice present increased neutrophil activation markers, adhesion, and rolling during endotoxemia (8). All of this evidence suggests that the Ang-(1-7)/MasR axis is an important negative regulator of neutrophil activation, leading to reduced migration to the inflamed tissue. Our experimental data are in line with the prior studies and show that preincubating neutrophils with Ang-(1-7) prevents cell migration toward the inflammatory stimulus (here, fMLP was used as the chemoattractant), potentially due to the neutrophil deactivation mechanisms described previously (8, 10, 11). On the other hand, Ang-(1-7) does not present any chemoattractant activity for neutrophils as it does for macrophages.

In immune cells, MasR is expressed in low levels (https://dice-database.org/genes/MAS1) but can be dynamically regulated by inflammatory stimuli, such as LPS (18). MasR activation was shown to be protective during a severe model of LPS-induced systemic inflammation and other preclinical models of inflammation (9, 18, 73, 74). Here, we have shown that MasR expression was upregulated during the resolution phase of inflammation, around the time regulatory macrophages are enriched in the pleural cavity. In addition, macrophages isolated at the resolution phase of *E*. *coli* peritonitis presented increased levels of MasR expression. Pharmacological inhibition of MasR delayed resolution of inflammation and recruitment of mononuclear cells, suggesting that MasR expression takes part in an endogenous program of self-resolving inflammation. Indeed, our data gathered from MasR^–/–^ mice reinforce an endogenous role for Ang-(1-7)/MasR in macrophage migration. In agreement with the role of Ang-(1-7)/MasR in the resolution of inflammation, dexamethasone, a drug that induces different features of resolution (35, 46), was shown to increase MasR expression (75). In sum, here we have identified, for the first time to our knowledge, the dynamic of MasR expression during resolving inflammation, implicating the receptor as a key player in this response. ACE-2 is the enzyme that catalyzes the conversion of Ang II to Ang-(1-7). Moreover, ACE-2 expression has been extensively mapped recently and shows that different cell types can potentially produce Ang-(1-7), including tissue macrophages in the context of inflammation (76). Indeed, ACE-2 was also shown to be upregulated by proinflammatory cytokines (77). Therefore, while the circulating physiological concentration of Ang-(1-7) is relatively low (78, 79), the local concentrations of Ang-(1-7), especially during inflammation, might be different. Endothelial and epithelial cells are also known to produce Ang-(1-7) (80, 81). Although our study has not investigated what specific cells are producing Ang-(1-7), we can suggest that in the context of inflammation-activated immune cells, epithelial and endothelial cells may be major producers of this peptide.

Besides macrophages and neutrophils, we do not rule out that the MasR/Ang-(1-7) pathway might also affect function of other cell types. Indeed, resident cells such as epithelial cells also express MasR (82) and can contribute to the removal of apoptotic neutrophils favoring restoration of tissue homeostasis (83). In both self-resolving models used here, MasR^–/–^ mice presented similar neutrophil number to WT, unlike the results obtained by the pharmacological inhibition of the receptor. One can hypothesize that the complete absence of MasR (MasR^–/–^) before and during a mild model of inflammation might be triggering resolution mechanisms for neutrophil removal, including efferocytosis by resident cells such as epithelial cells (83). In addition, the slight increase of macrophages observed in MasR^–/–^ at 8 hours (Figure 6E) or 12 hours (Figure 7D) postchallenge in both self-resolving models of inflammation might be sufficient to deal with the number of neutrophils observed in milder models of inflammation. Keeping with that, prior studies from our research group have shown that using the same self-resolving model of pleurisy, mice knocked out for GILZ, a proresolving protein induced by glucocorticoids, still resolved neutrophilic inflammation. Interestingly, this was associated with increased expression of annexin A1, another potent proresolving mediator (46). Similarly, annexin A1–knockout mice, a valuable tool to uncover annexin A1 proresolving mechanisms, can also resolve mild peritonitis elicited by zymosan at comparable rates to WT mice (84). Therefore, we believe similar mechanisms might be implicated here, and these will be further evaluated in future studies. In contrast to our mild model of pleurisy, increased and sustained numbers of neutrophils were observed in MasR^–/–^ mice, when compared with WT counterparts, after induction of a severe pleurisy (2.5 μg of LPS per cavity). Once again, macrophage numbers were significantly reduced in the early time points of pleural inflammation in MasR^–/–^ mice, while a rebound in the number of cells was observed at 48 hours post-LPS. Despite that, the efferocytosis ability of these late recruited MasR^–/–^ macrophages was substantially reduced when compared with WT cells, suggesting those cells are from proinflammatory phenotype, as reported in an EAE model (9). Therefore, neutrophil numbers were continuously increased in MasR^–/–^ mice at the WT resolution time point (48 hours). In agreement with our results from a severe model of pleurisy, MasR^–/–^ mice subjected to endotoxemia by intraperitoneal injection of higher amounts of LPS also presented a larger recruitment of neutrophils to the brain pia mater alongside increased levels of CXCL1 (8). Therefore, the pharmacological inhibition of MasR activation during inflammation, rather than before it has started, might be a more precise strategy for determining the role of this pathway during the resolution phase of mild models of inflammation as used in our work. Keeping with that, pharmacological inhibition of MasR at the peak of inflammation delayed resolution of neutrophilic inflammation in both of our inflammatory models.

Altogether, our data suggest the following mechanism for the newly recognized proresolving feature of Ang-(1-7): the Ang-(1-7)/MasR axis triggers the MEK/ERK1/2 pathway, leading to the production of CCL2 that, through a potential cooperation with MasR, induces CCR2-dependent recruitment of monocytes and polarization of macrophages toward a regulatory phenotype associated with increased production of IL-10 and TGF-β and efferocytosis (Figure 9). In addition, in the context of infection, Ang-(1-7)/MasR enhances macrophage migration and phagocytosis, aiding pathogen clearance. Therefore, the present study provides evidence that the Ang-(1-7)/MasR axis is a crucial pathway for the resolution of inflammation. Given the pivotal role of inflammation in the pathogenesis of COVID-19 and the involvement of ACE-2, the biosynthetic enzyme for Ang-(1-7), in viral biology, therapeutic strategies focusing on the modulatory actions of Ang-(1-7) are of interest (85–88). The identification of mechanisms and proresolving actions of Ang-(1-7) will pave the way for the development of host-targeted therapies for different inflammatory diseases.

## Methods

### BMDMs.

BM from tibias and femurs of C57BL/6 WT, CCR2^–/–^, or MasR^–/–^ mice was collected, and BMDMs were differentiated as previously described (89) and plated accordingly for different experiments (16).

### RAW 264.7 cell culture.

Murine macrophages (RAW 264.7 cells, obtained from the American Type Culture Collection) were serum deprived overnight and used for in vitro cell migration assays or Western blot analysis.

### Isolation of human PBMCs and neutrophils.

Peripheral blood from healthy donors was used for neutrophil isolation using the Histopaque gradient protocol (Histopaque 1077 and 1119 — MilliporeSigma) as previously described (28). Healthy donors were recruited in the University Hospital of Universidade Federal de Minas Gerais. PBMCs were obtained using the Ficoll-Paque PLUS protocol (GE Healthcare Bio-Sciences AB). Monocytes were isolated by immunomagnetic negative selection (EasySep Kit — StemCell Technologies).

### Ang-(1-7) source and purity.

Ang-(1-7) was purchased as a synthetic peptide from Bachem Inc., and purity (> 99%) was checked by the company using high-performance liquid chromatography. The peptide was diluted in endotoxin-free PBS, and solutions were tested by the *Limulus amebocyte* lysate endotoxin assay (Pierce). LPS contamination was insignificant (<0.5 endotoxin units/mL or <0.05 ng).

### In vitro cell migration experiments.

Macrophage chemotaxis assays were performed in 24-transwell plates (Corning) with polycarbonate membranes of pore size of 5.0 μm. Briefly, 5 × 10^5^ of RAW 264.7 BMDMs or human monocytes were added to the upper compartment of each well, while the chemoattractants [CCL2 or Ang-(1-7)] or media (control) were added to the lower compartments. Plates were incubated at 37°C for 4 hours when membranes were fixed and stained for cell counts. CCL2 (100 ng/mL) was used as a standard chemoattractant (positive control). In specific experiments, cells were preincubated for 1 hour with the CCR2 antagonist RS504393 (10 μM, Tocris Bioscience), the MasR antagonist A779 (1 μM, Bachem), the MEK/ERK inhibitors selumetinib (Selleck Chemicals) or U0126 (Cell Signaling Technology) (10 and 15 μM, respectively), or the vehicle of the drugs (DMSO 0.1%) and allowed to migrate toward Ang-(1-7) (100 nM). Similar experimental settings were utilized for cells plated onto 6-well plates (Corning, 1 × 10^6^ cells/mL) that were next harvested for Western blot analysis. In additional experiments, RAW 264.7 cells and BMDMs were pretreated with Ang-(1-7) (100 nM), and migration toward LPS (100 ng/mL) or control media was evaluated as mentioned above.

For neutrophil migration assays, 5 × 10^5^ cells were added to the upper parts of 24-transwell plates with polycarbonate membranes of pore size of 3.0 μm. A total of 100 nM of Ang-(1-7) was added to the bottom part of the plate, and cells were allowed to migrate for 4 hours at 37°C. In another set of experiments, neutrophils were pretreated with Ang-(1-7) (100 nM) 1 hour before the chemotaxis assay. In the latter experimental setup, RPMI (Cultilab) or fMLP (MilliporeSigma) (10^–9^ M in RPMI) was added to the lower compartments of the chamber. The system was incubated for 4 hours (37°C, 5% CO_2_), after which the membrane from the well inserts was removed, fixed, and stained for cell counts.

For both assays, leukocytes were counted under a light microscope (IX70 Olympus), and the average of counts in five 100× high-power fields was reported. Three technical replicates were performed, and the entire experiment was repeated at least 3 times.

### Migration of polarized macrophages.

To induce macrophage polarization to M1-like or M2-like phenotypes, BMDMs were exposed for 24 hours to mouse recombinant proteins as follows: IFN-γ (10 ng/mL) + LPS (10 ng/mL) to induce M1-like phenotype or IL-4 (20 ng/mL) to induce M2-like macrophages (16). M0 cells were exposed to complete RPMI only. Next, 5 × 10^5^ cells were transferred to 24-transwell plates, and the migration assay toward Ang-(1-7) was performed.

### In vitro production of cytokines.

BMDMs from WT C57BL/6 mice were washed out with RPMI without serum and later incubated with 100 nM of Ang-(1-7) or RPMI (untreated). At 6, 12, and 24 hours posttreatment, supernatant was harvested at for evaluation of IL-10 and TGF-β by ELISA. Supernatant CCL2 levels were evaluated at 4 hours post–Ang-(1-7) exposure.

### Ang-(1-7)–induced leukocyte migration in vivo.

Ang-(1-7) or PBS was locally injected in the pleura of BALB/c mice (100 ng/cavity). At 6, 24, and 48 hours postinjection, pleural lavage was performed to harvest the recruited leukocytes. Flow cytometry was used to validate microscopy findings at 48 hours postinjection. Next, pharmacological inhibition of CCR2, MasR, and the MEK/ERK pathway was achieved using RS504393 (2 mg/kg, i.pl.), A779 (200 ng/cavity, i.pl.) or U0126 (60 μg/cavity, i.pl.), respectively, 1 hour before Ang-(1-7) injection. Pleural lavages were performed, and recruited leukocytes were harvested for differential counting or Western blot analysis. CCL2 levels were evaluated by ELISA at different time points or at 6 hours (when inhibitors were used) post–Ang-(1-7) exposure.

Additionally, BALB/c mice received intra-articular injections of Ang-(1-7) (100 ng/cavity) or PBS into their tibiofemoral knee joint. At different time points, the articular cavity was washed 3 times with 5 μL of PBS for total and differential leukocyte counts. Periarticular tissue was removed for chemokine evaluation by ELISA.

### Models of LPS-induced pleurisy.

BALB/c and C57BL/6 WT or MasR-knockout (MasR^–/–^) mice (supplied in-house) were i.pl. injected with LPS (mild inflammation: 250 ng/cavity or severe inflammation: 2.5 μg/cavity) as described (16, 24). At 8, 24, and 48 hours post-LPS, pleural leukocytes were harvested from BALB/c challenged mice for total and differential cell counts and Western blot analysis of MasR expression. In addition, LPS-challenged BALB/c mice (250 ng/cavity) were locally treated with A779 (200 ng/cavity) or vehicle (PBS) at 8 and 24 hours post-LPS injections. Recruited leukocytes were analyzed at 48 hours post-LPS.

At 8 and 48 hours post-LPS, MasR^–/–^ and C57BL/6 WT mice were euthanized for leukocyte recruitment evaluation by microscopy (total and differential leukocyte counts and efferocytosis assessment) and flow cytometry.

### E. coli–induced peritonitis.

BALB/c and C57BL/6 WT or MasR-knockout (MasR^–/–^) mice were intraperitoneally infected with 1 × 10^6^ CFU of *E. coli* (ATCC 25922). At 6, 12, and 48 hours postinfection, peritoneal leukocytes were harvested for total and differential cell counts. Number of efferocytosis events was obtained by counting macrophages that ingested apoptotic neutrophils (500 cells/slide were counted) (24, 25). CCL2 levels were evaluated in the supernatants of the peritoneal lavages by ELISA. Bacterial loads were determined by plating the peritoneal lavage harvested at 6 hours postinfection in LB agar (USB Corporation) plates (37°C incubation, overnight). Western blot analysis was performed from macrophages harvested from PBS-injected or infected mice at 12 and 48 hours post–*E*. *coli* by adherence exclusion.

### Resolution intervals for E. coli peritonitis.

BALB/c mice were intraperitoneally infected with 1 × 10^6^ CFU of *E. coli* (ATCC 25922) and were treated with A779 (200 ng/cavity) or vehicle (PBS) at 12 hours and 24 hours postinfection. Resolution intervals were calculated by determining the time interval from the maximum neutrophil numbers to the 50% reduction point.

### Phagocytosis assays.

Phagocytosis was evaluated as previously done (90). Briefly, 2 × 10^5^ peritoneal macrophages or BMDMs isolated from naive mice were plated and incubated with *E*. *coli* (MOI 1:10) for 3 hours to allow phagocytosis (1 hour of adhesion at 4°C followed by 2 hours at 37°C). Noninternalized bacteria were washed out with gentamycin (Gentatec, Chemitec; 5 μg/mL in PBS, 30 minutes). To assess phagocytosed bacteria, macrophages were lysed as described (90), and viable internalized bacteria were counted in LB agar plates after incubation (37°C, overnight). For the killing assay, macrophages were incubated for an extra 2 hours postincubation with gentamycin; then cells were lysed and viable bacteria were counted as described before (70).

### Flow cytometry.

Leukocytes were stained with the following antibodies: F4/80 (BM8)-PEcy7 (BioLegend), Ly6C (AL-21)-APCCy7, Ly6G (1A-8)-APC, CD11b (M1/70)-BV421, CD206 (19.2)-APC, and CD3 (SK7)-BV421 (BD Biosciences). Total macrophages (F4/80^+^), monocytes (Ly6C^+^F4/80^–^), neutrophils (Ly6G^+^), and lymphocytes (CD3^+^) were evaluated. Events were acquired in FACSCanto II (BD Biosciences) and analyzed using FlowJo Software (Tree Star Inc.). Macrophage phenotypes were defined by the expression of F4/80, CD11b, Ly6C, and CD206, as described previously (16, 25).

### ELISA.

Levels of IL-10, TGF-β, CCL2, CXCL1, TNF-α, and IL-6 were measured in the supernatants obtained from pleural and peritoneal lavages, cell culture–free supernatants and/or periarticular tissue homogenates. ELISA was performed using commercially available antibodies according to the procedures supplied by the manufacturer (R&D Systems).

### Western blotting.

Western blot was performed as previously done (25, 26). Samples were electrophoresed on denaturing 10% polyacrylamide SDS gels followed by transfer to nitrocellulose membranes. Membranes were blocked for 1 hour with 5% of nonfat dry milk solution (PBS 0.1% Tween-20) and incubated overnight with anti–p-ERK1/2 (1:1000 — 4377-197G2, Cell Signaling Technology), anti-MasR (MAS1L 1:500 — ab200685 Abcam), anti–Arg-1 (1:1000, sc-20150 — H52, Santa Cruz Biotechnology), anti-Ym1 (60130, 1:1000 — StemCell Technologies), or anti–β-actin (1:3000, A5316 – AC-74, MilliporeSigma). Secondary anti-rabbit (7074, Cell Signaling Technology) or anti-mouse (sc-2005, Santa Cruz Biotechnology) HRP-conjugated antibodies (1:3000) were added to the membranes for further incubation of 1 hour at room temperature. ECL detection system (GE Healthcare, now Cytiva) was used to visualize immunoreactive bands. Membranes were scanned and densitometry analysis of the bands was performed using ImageJ software. Results were expressed as arbitrary units and normalized using β-actin levels as loading controls.

### Immunofluorescence.

Cells were fixed (2% buffered paraformaldehyde) 48 hours post–Ang-(1-7) injection (i.pl. 100 ng/cavity), washed, and permeabilized (0.5% Triton X-100 in PBS). Nonspecific antibody bindings were prevented by blocking the samples with nonimmune 5% goat serum and 5 μg/mL mouse BD Biosciences Fc Block. Primary antibodies — PE-conjugated anti-CCR2 (LS132.1D9 1:50 — R&D Systems), APC-conjugated rat anti-F4/80 (T45-2342 1:50 — BD Biosciences), and rabbit anti-MasR (MAS1L ab200685 1:200 — Abcam) — were incubated overnight at 4°C. Negative controls were established by omitting primary antibodies. After washing, cells were exposed for 1 hour at room temperature to the Alexa Fluor 488–conjugated goat anti-rabbit secondary antibody (ab150077 1:100 — Abcam) and stained with DAPI (1 μg/mL, MilliporeSigma). The fluorescent signals were evaluated using an inverted Nikon Eclipse Ti confocal microscope coupled to an A1 scanning head. For each sample, the percentage of F4/80^+^ macrophages expressing CCR2 and MasR was estimated in 10 randomly selected pictures at 200× original magnification by using the Nikon NIS-Elements cell counter.

### Efferocytosis assay.

Isolated neutrophils from peripheral blood of healthy donors were incubated with 10 μM of staurosporine (MilliporeSigma) for 1 hour to induce apoptosis (verified by flow cytometry using annexin V-FITC and propidium iodide staining). Apoptotic neutrophils were labeled with 5 μM CFSE (37°C and 5% CO_2_ for 1 hour). Next, WT and MasR^–/–^ BMDMs were coincubated for 1 hour with CFSE-labeled neutrophils in a proportion of 3 apoptotic neutrophil:1 macrophage). Flow cytometry was performed and efferocytosis was evaluated (MFI of CFSE^+^ cells in F4/80^+^) (16, 25).

Efferocytosis was also assessed as previously described (24, 25). At 71 hours post-zymosan injections (0.1 mg/mouse, i.p.), WT and MasR^–/–^ mice received 3 × 10^6^ apoptotic human neutrophils intraperitoneally. One hour later, peritoneal leukocytes were harvested for efferocytosis quantification by microscopy analysis of cytospin slides (500 cells/slide were counted). Results are expressed as the frequency of macrophages that ingested apoptotic neutrophils.

### Data availability.

Data sets generated are available in the current manuscript and supplemental materials file.

### Statistics.

Data were analyzed by 1-way ANOVA, followed by the Newman-Keuls test, or 2-way ANOVA, followed by Bonferroni’s multiple comparisons test (difference between backgrounds) or Tukey’s multiple comparisons test (differences between time points). When only 2 groups were evaluated, a 2-tailed *t* test was used. A value of *P* < 0.05 was considered significant. Results were presented as mean ± SEM. Statistical calculations were performed using GraphPad Prism 6.0.

### Study approval.

Experiments had prior approval from the Animal Ethics Committee of Universidade Federal de Minas Gerais (UFMG) (CEUA, protocol number: 295/2018) and Research Ethics Committee of UFMG, for human cell studies (COEP, protocol number 0319.0.203.000-11). Male BALB/c mice (8–10 weeks) obtained from the local animal facility were maintained under standard housing conditions. C57BL/6 WT mice, MasR-knockout mice (MasR^–/–^, generated as previously described in ref. 91), and CCR2-knockout mice (CCR2^–/–^, generated as described in ref. 19) were bred and maintained at the local animal facility at UFMG.

## Author contributions

LPS, IZ, MAS, IG, and LPT analyzed data and wrote the paper. IZ, MAS, KML, GLNL, LCRT, BVSV, AC, JPV, GHCS, JAMS, TCM, and LCG performed the experiments and analyzed data. VP, MJCS, RASS, and MMT provided expertise and improvements and helped with paper discussion. RASS and MJCS provided the MasR-knockout mice. LPS designed research. LPT and IZ have contributed equally to the technical and scientific aspects of the work. All authors have read and agreed to the published version of the manuscript.

## Supplementary Material

Supplemental data

## Version 1 12/07/2021

In-Press Preview

## Version 2 01/11/2022

Electronic publication
