# Supplementary material for: Angiotensin-(1-7)/MasR axis promotes migration of monocytes/macrophages with a regulatory phenotype to perform phagocytosis and efferocytosis
Source: JCI Insight. 2022 Jan 11;7(1):e147819. doi: 10.1172/jci.insight.147819 (PMC8765051; doi:10.1172/jci.insight.147819)
Supplement: Supplemental data [file jciinsight-7-147819-s010.pdf]

Supplementary Figures:

Supplementary Figure 1

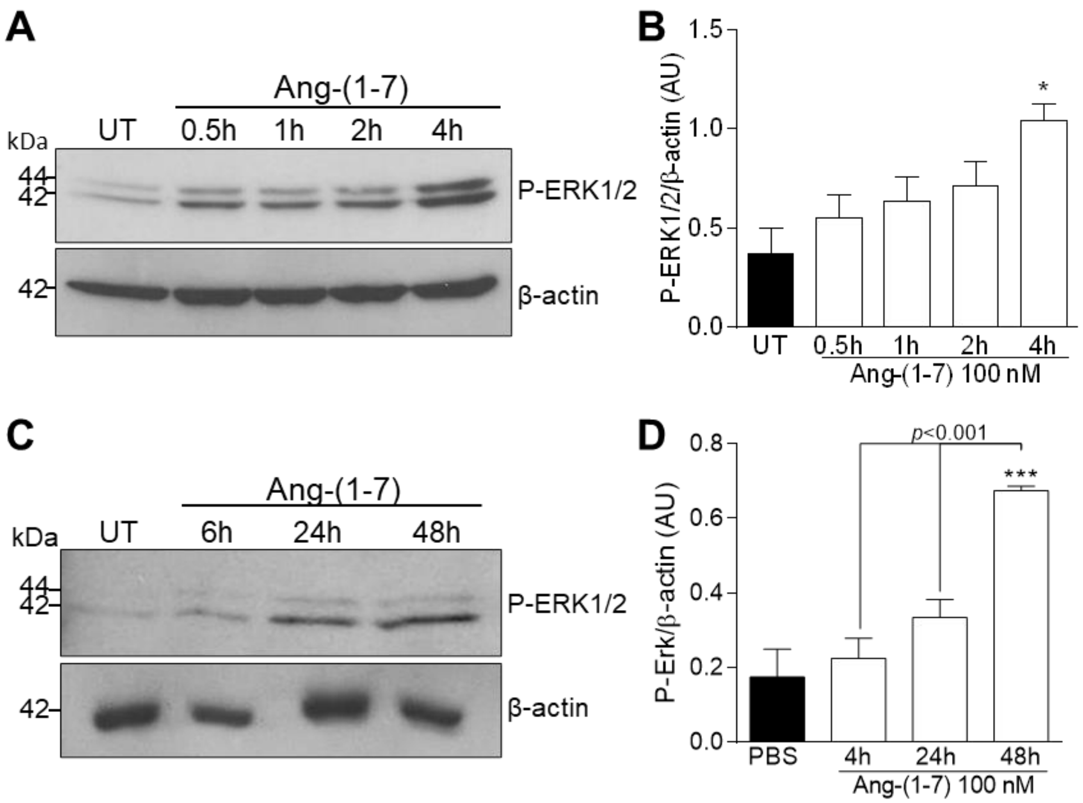

Supplementary Figure 1. Ang-(1-7) exposure in vitro and in vivo increases ERK1/2 phosphorylation. RAW 264.7 cells were incubated with 100nM of Ang-(1-7) or RPMI (UT) and harvested at 0.5, 1, 2 and 4 h post-exposure. Western blot analysis was performed to evaluate P-ERK1/2 (A-B). In another experiment, WT BALB/c mice were intrapleurally injected with 100ng of Ang-(1-7) and euthanized at, 24h and 48h post-injection. Lysates of pleural lavage leukocytes were used to quantify P-ERK1/2 by Western Blot (C-D). β-actin was used as protein loading control. Quantification was performed using the ImageJ Software (B-D). Data is presented as the mean ± SEM (n= 3) , * for p<0.05 and *** for p<0.001 when compared to controls (PBS) by one-way ANOVA.

Supplementary Figure 2

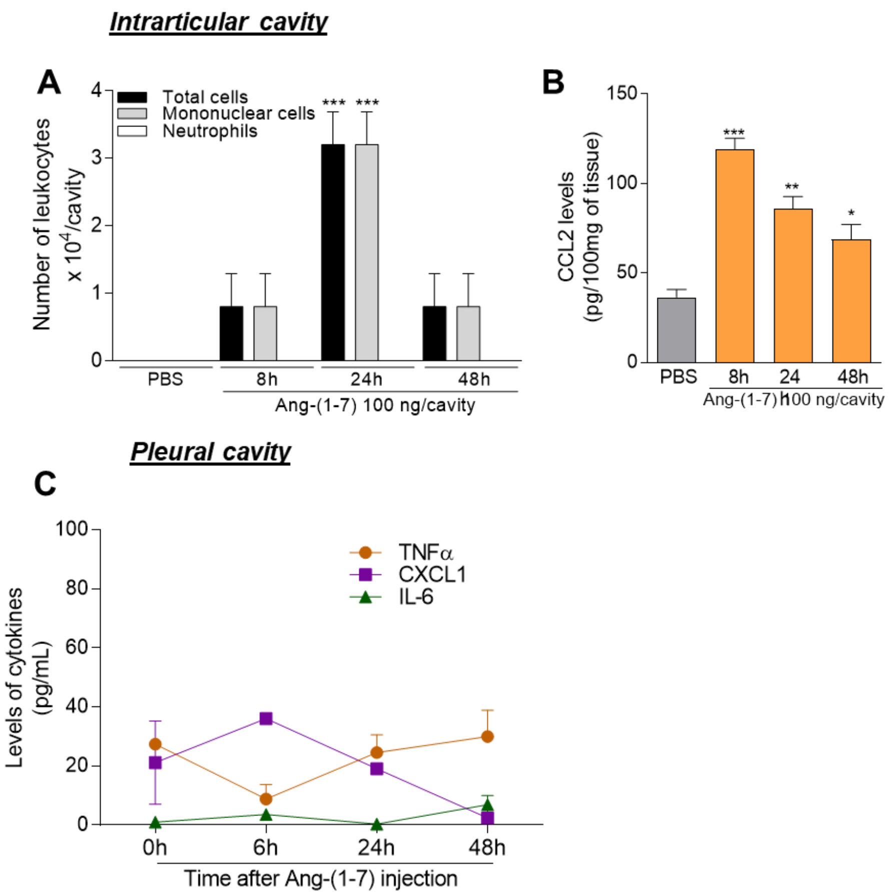

Supplementary Figure 2. Ang-(1-7) promotes mononuclear cell recruitment and CCL2 release without production of inflammatory cytokines. Ang-(1-7)-100ng/mouse or PBS were injected into the intraarticular cavity of WT BALB/c mice. At 8, 24 and 48h post-injection, recruited leukocytes were harvest for differential counts (A) and periarticular tissue was used for CCL2 measurements (B). In another experiment, Ang-(1-7) was injected in the pleural cavity of mice and levels of TNF-α, CXCL-1 and IL-6 were evaluated in pleural lavage supernatants (C). Results are shown as the mean ± SEM of 5 mice in each group. * for $p<0.05$, ** for $p<0.01$ and *** for $p<0.001$ when compared to PBS-injected mice by one-way ANOVA.

Supplementary Figure 3

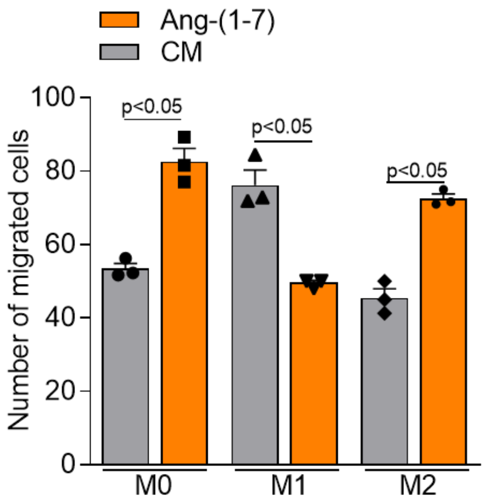

Supplementary Figure 3. Ang-(1-7)-induced migration of polarized macrophages. WT BMDMs (M0) were maintained with medium without serum or polarized to M1 and M2-like macrophages (see methods), and chemotaxisassays were performed using 100nM of Ang-(1-7) as the chemoattractant agent. RPMI was used as a negative control (control medium). After 4 hours of migration, polycarbonate membranes were harvested, fixed, and stained with hematoxylin for cell counts under light microscopy. Results are shown as the mean ± SEM of the absolute number of migrated cells and differences were calculated by one-way ANOVA.

Supplementary Figure 4

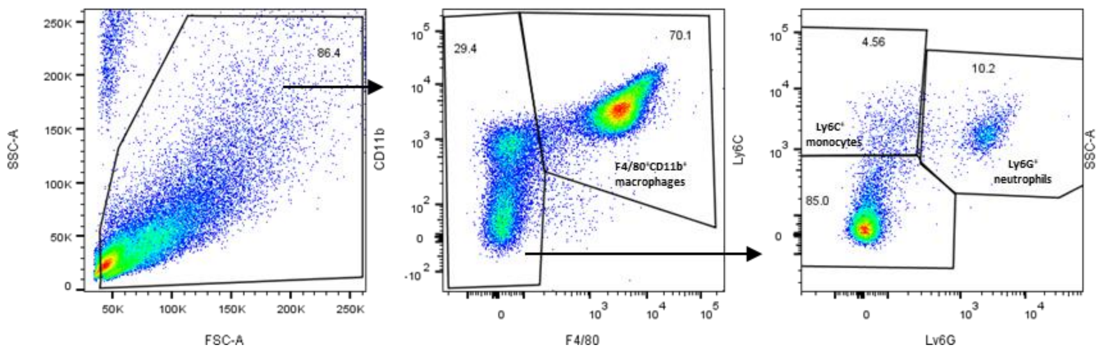

Supplementary Figure 4. Gating strategy for flow cytometry analysis of pleural leukocytes. Briefly, total cells were first gated based on the FSC-A and SSC-A to exclude debris, and the subpopulations of the indicated leukocytes were gated based on specific surface markers as indicated in the individual panels. Macrophages were identified by the expression of F4/80 and CD11b (F4/80+CD11b+ cells). Next, monocytes (Ly6C+) and neutrophils (Ly6G+) were identified. Values inside the plots represent the percentages from the parent gate. SSC-A: side scatter area, FSC-A: forward scatter area.

Supplementary Figure 5

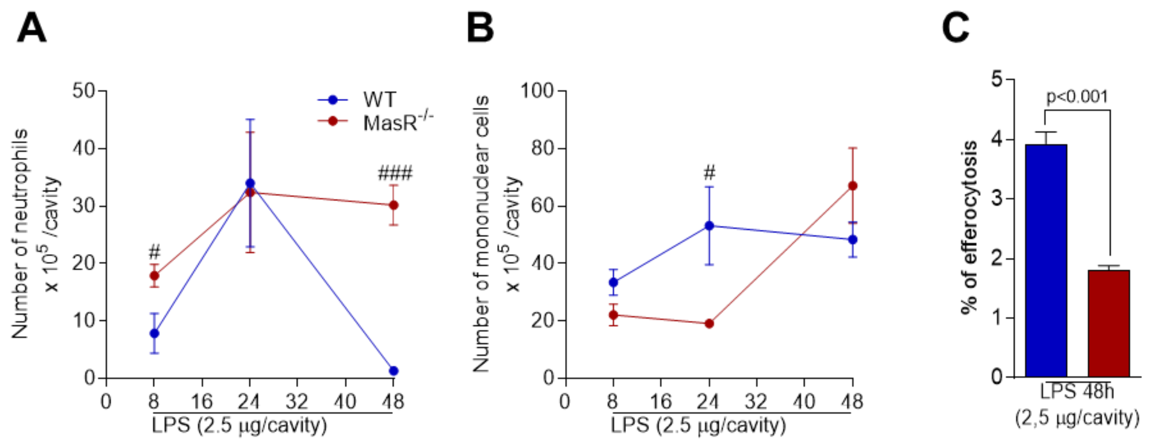

Supplementary Figure 5. MasR mice have increased neutrophilic inflammation, early reduced recruitment of macrophages and diminished efferocytosis. BALB/c mice were challenged with LPS (2.5µ/cavity, i.pl.) or PBS and leukocytes from the pleural cavity were harvest after 8, 24 and 48h for neutrophil (A) and mononuclear cell counts (B). The frequency of efferocytosis was evaluated at 48 post-LPS by morphological identification in cytospin slides (C). Results are shown as the mean ± SEM of at least 5 mice in each group. # for $p<0.05$, and ### for $p<0.001$ by one-way ANOVA followed by Holm-Šídák's multiple comparisons test (A,B) or T-test (C).

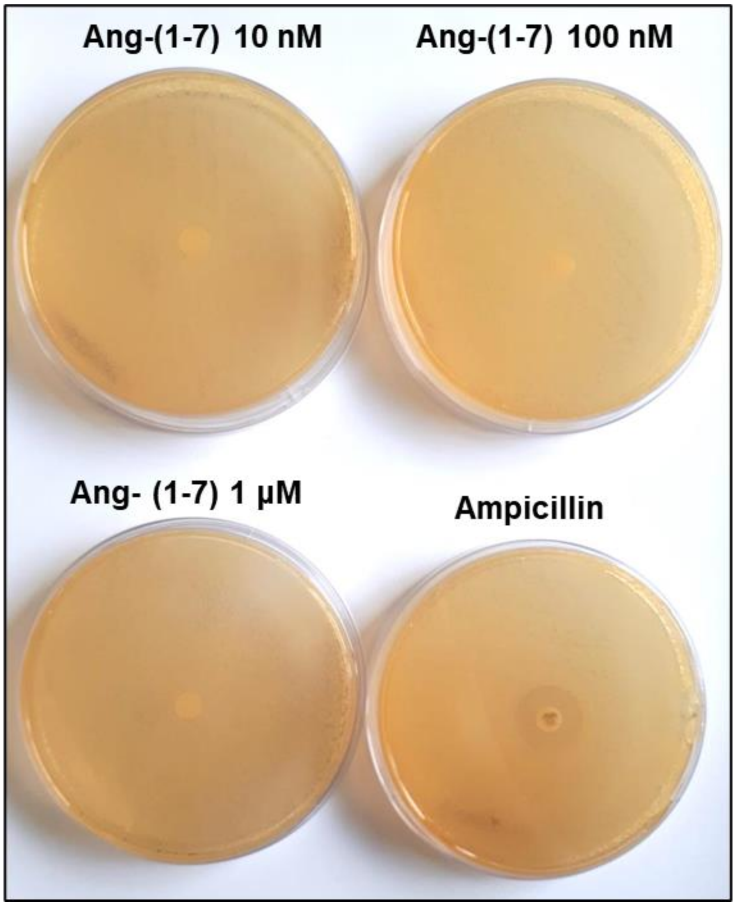

Supplementary Figure 6. Ang-(1-7) does not display direct antibacterial actions. Ang-(1-7) (10nM-1µm) or ampicillin (control) were placed on sterile filters that were then added to Mueller Hinton agar plates containing E. coli. The zone of growth inhibition was evaluated after overnight incubation at 37 ^{o}C.

Supplementary Figure 7

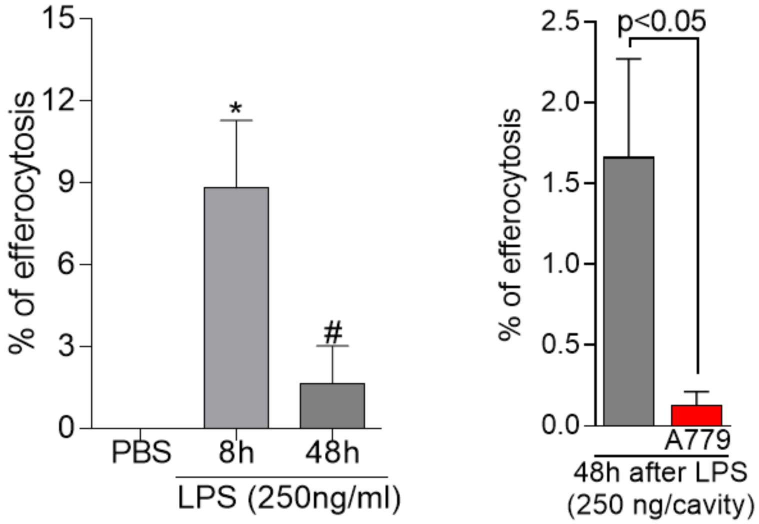

Supplementary Figure 7. Efferocytosis evaluation during LPS-induced inflammation. BALB/c mice were challenged with LPS (250ng/cavity, i.pl.) or PBS. Efferocytosis was morphologically identified in cytospin slides stained with May-Grunwald-Giemsa and % of efferocytosis was evaluated by counting 500 cells per slide (A). In another experiment, mice were treated with A799 (200ng/cavity) or vehicle (PBS) at 8 and 24h after LPS challenge (250ng/cavity). At 48h post-LPS, pleural lavages were performed, and efferocytosis was assessed as above mentioned. Results are shown as the mean ± SEM of at least 5 mice in each group. *p<0.05 when compared with PBS-injected mice and # p<0.05 when compared to 8h LPS-injected mice, by one-way ANOVA (A) or t-test (B).
